# Renal Angptl4 is a key fibrogenic molecule in progressive diabetic kidney disease

**DOI:** 10.1126/sciadv.adn6068

**Published:** 2024-12-04

**Authors:** Swayam Prakash Srivastava, Han Zhou, Rachel Shenoi, Myshal Morris, Begoña Lainez-Mas, Leigh Goedeke, Barani Kumar Rajendran, Ocean Setia, Binod Aryal, Keizo Kanasaki, Daisuke Koya, Ken Inoki, Alan Dardik, Thomas Bell, Carlos Fernández-Hernando, Gerald I. Shulman, Julie E. Goodwin

**Affiliations:** ^1^ Department of Pediatrics, Yale University School of Medicine, New Haven, CT, USA.; ^2^ Vascular Biology and Therapeutics Program, Yale University, New Haven, CT, USA.; ^3^ Life Sciences Institute, University of Michigan, Ann Arbor, MI, USA.; ^4^ Department of Diabetology and Endocrinology, Kanazawa Medical University, Uchinada, Japan.; ^5^ Department of Medicine, Yale University School of Medicine, New Haven, CT, USA.; ^6^ Department of Medicine (Cardiology), The Cardiovascular Research Institute, Icahn School of Medicine at Mount Sinai, New York, NY, USA.; ^7^ Department of Pharmacology, Yale University School of Medicine, New Haven, CT, USA.; ^8^ Department of Surgery, Yale University School of Medicine, New Haven, CT, USA.; ^9^ Department of Surgery, VA Connecticut Healthcare Systems, West Haven, CT, USA.; ^10^ Department of Comparative Medicine, Yale University School of Medicine, New Haven, CT, USA.; ^11^ Department of Internal Medicine 1, Shimane University Faculty of Medicine, 89-1 Enya-cho, Izumo, Shimane 693-8501, Japan.; ^12^ The Center for Integrated Kidney Research and Advance, Faculty of Medicine, Shimane University, 89-1 Enya-cho, Izumo 693-8501, Japan.; ^13^ Department of Molecular and Integrative Physiology, University of Michigan Medical School, Ann Arbor, MI, USA.; ^14^ Department of Internal Medicine, Division of Nephrology, University of Michigan Medical School, Ann Arbor, MI, USA.; ^15^ Ionis Pharmaceuticals, Carlsbad, CA, USA.; ^16^ Department of Pathology, Yale University School of Medicine, New Haven, CT, USA.; ^17^ Department of Cellular and Molecular Physiology, Yale University School of Medicine, New Haven, CT, USA.

## Abstract

Angiopoietin-like 4 (ANGPTL4), a key protein involved in lipoprotein metabolism, has diverse effects. There is an association between Angptl4 and diabetic kidney disease; however, this association has not been well investigated. We show that both podocyte- and tubule-specific ANGPTL4 are crucial fibrogenic molecules in diabetes. Diabetes accelerates the fibrogenic phenotype in control mice but not in ANGPTL4 mutant mice. The protective effect observed in ANGPTL4 mutant mice is correlated with a reduction in stimulator of interferon genes pathway activation, expression of pro-inflammatory cytokines, reduced epithelial-to-mesenchymal transition and endothelial-to-mesenchymal transition, lessened mitochondrial damage, and increased fatty acid oxidation. Mechanistically, we demonstrate that podocyte- or tubule-secreted *Angptl4* interacts with Integrin β1 and influences the association between dipeptidyl-4 with Integrin β1. We demonstrate the utility of a targeted pharmacologic therapy that specifically inhibits *Angptl4* gene expression in the kidneys and protects diabetic kidneys from proteinuria and fibrosis. Together, these data demonstrate that podocyte- and tubule-derived *Angptl4* is fibrogenic in diabetic kidneys.

## Teaser

Angplt4 deficiency in podocytes and renal tubule cells is protective in diabetes.

## INTRODUCTION

About one-third of patients with diabetes develop diabetic kidney disease (DKD), which is a leading cause of end-stage renal disease (*1*, *2*). Kidney fibrosis is the final consequence of DKD (*3*, *4*). The molecular pathways that link renal fibrosis and cardiovascular disease, two key components of diabetic nephropathy, are not well known (*2*, *5*, *6*). This knowledge gap contributes to the suboptimal treatment options available for these patients. Mechanisms causing renal fibrosis include immune cell activation, pro-inflammatory cytokines, aberrant levels of chemokines, tubular cell apoptosis, endothelial cell dysfunction, podocyte cell dysfunction, and mesenchymal activation in diverse kidney cell types (*7*–*13*). A recent study of spatial transcriptomics in human kidney identified four distinct cellular microenvironments: glomerular, immune, tubule, and fibrotic, and provided a comprehensive spatially resolved molecular roadmap of the human kidney and the fibrotic process, demonstrating the importance of cell specificity in the development of fibrosis (*14*).

Although there are controversial hypotheses about myofibroblast origins, such as from epithelial cells via epithelial-to-mesenchymal transition (EMT), from endothelial cells via endothelial-to-mesenchymal transition (EndMT), from macrophages via macrophage-to-mesenchymal transition, and from resident fibrocytes from bone marrow origin, these myofibroblasts are characterized by the expression of mesenchymal markers (α-smooth muscle actin, N-cadherin, and vimentin) and contribute to organ fibrogenesis (*6*, *9*, *15*–*21*). Intermediate cell types may influence mesenchymal activation processes and the health of neighboring cells (*20*, *22*–*25*). Biological pathways such as activated transforming growth factor-β (TGFβ) signaling (*26*, *27*), Notch signaling (*28*–*30*), Wnt signaling (*31*–*34*), and Hedgehog signaling (*35*–*37*) lead to disruption in central metabolism and cause mesenchymal metabolic shifts in diabetic kidneys, ultimately resulting in deposition of collagens, extracellular matrix proteins, fibronectin, vimentin, and N-cadherin in interstitial spaces (*38*, *39*). Angiopoietin-like 4 (ANGPTL4) is known to inactivate lipoprotein lipase (LPL) activity and is expressed primarily in adipose tissues, liver, and skeletal muscle (*40*–*42*). A major factor determining plasma triglyceride (TG) levels is endothelial-bound LPL (*40*, *42*), which hydrolyzes TG, releases nonesterified fatty acids, and also promotes tissue uptake of nonesterified fatty acids (*40*, *42*). Clement *et al.* (*43*) described two forms of ANGPTL4: a high-isoelectric point pro-proteinuric form that is found only in glomeruli and is hyposialylated, and a sialylated, neutral isoelectric point form that is secreted from adipose tissue, liver, and skeletal muscle. Conversion of the hyposialylated form to the sialylated form by treatment with *N*-acetyl-d-mannosamine suppresses proteinuria and the disease phenotype in a mouse model of nephrotic syndrome (*43*). Studies using a podocyte-specific transgenic overexpression model that has higher podocyte-specific Angptl4 secretion demonstrated more proteinuria (~500-fold higher), loss of glomerular basement membrane (GBM) charge, and foot process effacement, whereas transgenic overexpression in adipose tissue resulted in increased circulating Angptl4, but no proteinuria, suggesting opposing effects of Angptl4 in renal tissues as compared to extrarenal tissues (*43*, *44*). The increase in circulating Angptl4 in response to nephrotic-range proteinuria reduces the severity of the disease phenotype; however, it induces hypertriglyceridemia, suggesting a potential link between circulatory Angplt4 with proteinuria and dyslipidemia in nephrotic syndrome (*44*, *45*). Of note, the CD-1; *db/db* mouse, which is a type 2 diabetic mouse model that mimics the renal fibrotic features of human DKD, has significantly higher levels of kidney *Angplt4* gene expression, suggesting a potential role of Angplt4 in DKD (*46*).

Metabolic reprogramming, which is characterized by alterations in fuel preference in different cell types, is one the predominant features of mesenchymal activation in injured kidney cells (*16*, *33*, *47*–*51*). It is still not well understood how these metabolic alterations are regulated or how they stimulate proliferation of mesenchymal cells. However, it has been shown that suppression of fatty acid oxidation (FAO) in injured epithelial cells is involved in the development of fibrosis and that abnormal glycolysis in injured endothelial cells contributes to mesenchymal activation processes (*16*, *47*, *48*, *52*, *53*). Such metabolic switching is key to understanding fibrogenic programs. Genetic deficiency of Angptl4 improves glucose homeostasis and diabetes (*54*) and hepatocyte-deficient mutant mice demonstrate improved hepatocyte FAO and associated improvements in obesity, diabetes, and atherosclerosis (*55*). Here, our results show the crucial role of both podocyte- and tubule-specific Angptl4 in the pathogenesis of DKD. Targeted inhibition of kidney-specific Angptl4 is a promising therapeutic option for the management of DKD.

## RESULTS

### Loss of Angptl4 protects against a fibrotic phenotype in a murine model of progressive DKD

**Fig. 1.**
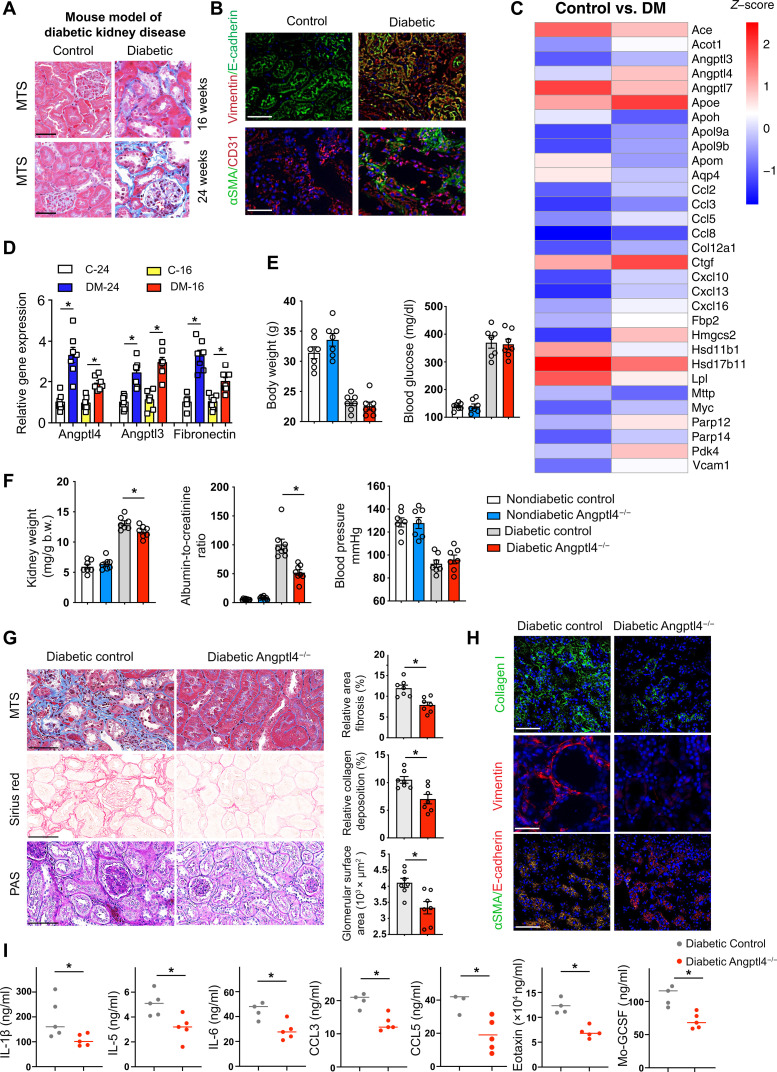
Up-regulated Angptl4 expression is associated with DKD. (**A**) Masson trichrome staining (MTS) in kidneys of male CD-1 mice. Scale bar, 50 μm. (**B**) Immunofluorescence analysis of vimentin/E-cadherin and αSMA/CD31 in kidneys from nondiabetic and diabetic mice. FITC-labeled E-cadherin, rhodamine-labeled vimentin, and DAPI (nuclei, blue); FITC-labeled αSMA, rhodamine-labeled CD31, and DAPI (nuclei, blue). Representative merged (original magnification 400×) images are shown. Scale bar, 50 μm. (**C**) mRNA array analysis in control and diabetic mice. Heatmap of analyzed gene expression. (**D**) Relative gene expression levels of *Angptl4*, *Angplt3*, and *fibronectin* in control and diabetic mice. *n* = 7 mice per group. (**E** and **F**) Physiological characteristics in diabetic and nondiabetic mice of both genotypes. *n* = 7 mice per group except for blood pressure where *n* = 6 mice per group. Two replicate experiments were analyzed. Data are mean ± SEM. One-way ANOVA with Tukey’s multiple comparison test was used to calculate statistical significance. (**G**) Histologic images of MTS, Sirius red, and PAS staining in diabetic control and Angptl4^−/−^ mice (original magnification, 300×). Quantification was performed using ImageJ. Representative images are shown. Scale bar, 100 μm. *n* = 7 mice per group. (**H**) Immunofluorescence analysis of collagen I, Vimentin, and αSMA/CD31 was performed in kidneys from diabetic control and diabetic Angptl4^−/−^ mice. FITC-labeled collagen I, rhodamine-labeled vimentin, FITC-labeled αSMA, rhodamine-labeled E-cadherin, and DAPI (nuclei, blue) were used. Representative merged (original magnification, 200×) images are shown. Scale bar, 50 μm. *n* = 7 mice per group. Three independent experiments were analyzed. (**I**) Plasma levels of IL-1β, IL-5, IL-6, CCL3, CCL5, eotaxin, and Mo-GCSF were analyzed by cytokine array analysis. *n* = 3 to 5 control mice and *n* = 5 Angptl4^−/−^ mice. Data are mean ± SEM. Student’s *t* test (unpaired two tailed) was used for analysis of statistical significance. Significance: *P < 0.05.

Streptozotocin (STZ)–induced diabetic mice are the established mouse model for studying DKD. Diabetes mellitus caused gain of time-dependent fibrogenic kidney phenotypes, a higher rate of EMT, as evidenced by higher vimentin expression in E-cadherin–positive cells, and a higher rate of EndMT, as shown by higher αSMA in CD31-positive cells, at 24 weeks after STZ injection, when compared to nondiabetic control mice (Fig. 1, A and B). To study cellular changes more broadly in DKD, we performed mRNA array analysis in control and fibrotic diabetic kidneys. Array data demonstrate that there were 284 genes up-regulated (>2-fold) and 150 genes down-regulated (>0.5-fold) in diabetes, and these altered genes were associated with fibrosis-related biological functions and pathways (Fig. 1C and figs. S1 and S2). We found significant alteration in the expression of genes regulating LPL. LPL was significantly down-regulated and the LPL regulators *Angptl3* and *Angplt4* were significantly up-regulated in diabetes (Fig. 1C). To test the role of LPL in the phenotype of DKD, we used a chemical inhibitor of LPL, poloxamer 407, in diabetic mice that have early-onset kidney fibrosis. Poloxamer 407 treatment suppressed LPL activity and increased the level of plasma TGs. However, it did not change the severity of kidney fibrosis when compared to untreated mice, suggesting that suppression in LPL activity is not related to fibrosis in diabetic kidneys (fig. S3). The mRNA expression levels of the LPL regulators, *Angptl3* and *Angplt4*, were higher in time-dependent fibrotic kidneys, similar to the pattern observed in fibronectin. *Angptl3* and *Angplt4* mRNA expression was found to be higher in high-glucose (30 mM)–treated cultured human tubular HK-2 cells than in low-glucose conditions (Fig. 1D and fig. S4A). *Angptl4* knockdown abolished the TGFβ1-associated expression of fibrogenic genes in high-glucose–stimulated cells while *Angptl3* did not have such a prominent effect, suggesting that *Angptl4* is a key contributor to fibrogenesis in tubule cells (fig. S4B).

The metabolic function of *Angptl4* in regulating proteinuria and its relationship with hypertriglyceridemia in a mouse model of glomerular disease have been described previously (*43*, *44*). *Angptl4* is highly expressed in the human kidney (fig. S4C), although its function in regulating metabolism in diabetes mellitus is poorly understood. Reviewing human data from a publicly available human atlas (https://proteinatlas.org/), it is evident that *Angptl4* is expressed in glomerular cells and tubules in both males and females. To investigate the role of *Angplt4* in DKD, we used Angptl4 mutant (Angplt4^−/−^) mice. Angptl4^−/−^ mice and littermate controls were born in expected Mendelian ratios. Angptl4^−/−^ mice were generated by the knockout-first strategy (*56*). These mice have a cassette containing the mouse En2 splicing acceptor, LacZ, and promoter-driven neomycin resistance gene and were inserted in the *Angptl4* gene. The initial allele (tm1a) generates a null allele through splicing to the LacZ trapping element. Because the expression of the LacZ reporter gene is regulated by the *ANGPTL4* promoter gene in the original tm1a mice, the expression of ANGPTL4 was detected using an antibody against β-galactosidase (*57*). Angptl4^−/−^ mice kidneys had higher protein expression of β-gal as compared to littermate controls (fig. S5A). Diabetes was induced in control and Angptl4^−/−^ mice with STZ for 20 weeks. We did not observe any remarkable differences in body weight, blood glucose, kidney weight, albumin-to-creatinine ratio, or blood pressure in the nondiabetic mice. Blood glucose, kidney weight, and albumin-to-creatinine ratios were significantly higher in diabetic control mice. Blood pressure was significantly lower in diabetic mice of both genotypes, as expected (Fig. 1, E and F). There were no differences in fibrosis levels between the nondiabetic control and Angplt4^−/−^ mice (fig. S5B). Diabetes increased the area of fibrosis, relative collagen deposition, and extent of glomerulosclerosis in the kidneys of control mice; however, the same effect was not observed in the kidneys of Angplt4^−/−^ mice (Fig. 1G and fig. S5C). The mRNA expression of α*SMA* and *COL1A1* were significantly down-regulated in diabetic Angplt4^−/−^ mice (fig. S5D). Immunofluorescence analysis revealed higher collagen I deposition and vimentin levels in kidneys from diabetic control mice when compared to kidneys from diabetic Angplt4^−/−^ mice. Diabetes enhanced the rate of EMT in the kidneys of control mice as evidenced by increased colocalization of αSMA in E-cadherin–positive cells; this effect was not prominent in kidneys from Angplt4^−/−^ mice (Fig. 1H). Aberrations in cytokine and chemokine levels are associated with mesenchymal transformation in tubules and endothelial cells; therefore, plasma cytokine levels were measured. Diabetic Angplt4^−/−^ mice had suppressed levels of pro-inflammatory cytokines and chemokines, such as IL-1β, IL-5, IL-6, CCL3, CCL5, eotaxin, and Mo-GCSF, when compared to diabetic control mice (Fig. 1I).

The phenotype of the Angplt4^−/−^ mice was also evaluated using an accelerated model of nephropathy, which combined the effects of both the STZ-induced diabetic model as well as the unilateral urinary obstruction (UUO) model (*34*). In this model, control and Angplt4^−/−^ mice were subjected to UUO followed by four daily doses of STZ and euthanasia on day 12. There were no differences in blood glucose concentrations between the two groups but UUO-operated Angplt4^−/−^ mice demonstrated lower kidney weights and lessened fibrosis when compared to UUO-operated control mice (fig. S6, A and B).

### Metabolic reprogramming by Angptl4 deficiency protects against DKD

**Fig. 2.**
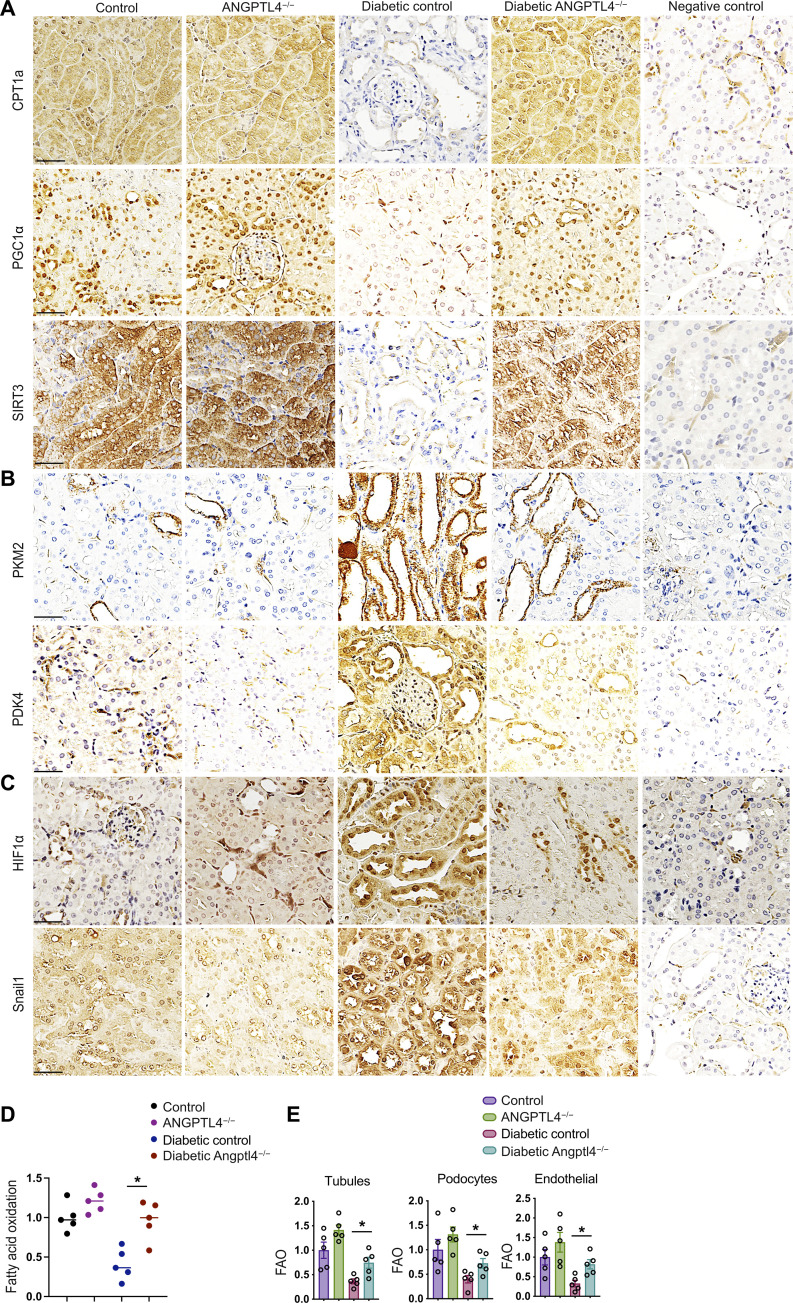
Metabolic reprogramming by Angptl4 deficiency protects against DKD. (**A** to **C**) Immunohistochemical analysis of CPT1a, PGC1α, SIRT3, PKM2, PDK4, HIF1α, and Snail1 expression in kidneys from nondiabetic and diabetic, control, and Angptl4^−/−^ mice. Scale bar, 50 μm. *n* = 6 mice per group. Two independent replicate experiments were performed. Representative micrographic images (original magnification, 300×) are shown. (**D**) Radiolabeled [^14^C]palmitate oxidation and [^14^CO_2_] release were measured. CPM of each sample was counted. *n* = 5 per group. (**E**) Fatty acid oxidation analysis in isolated tubules, podocytes, and endothelial cells from nondiabetic and diabetic, control, and Angptl4^−/−^ mice. Radiolabeled [^14^C]palmitate oxidation and [^14^CO_2_] release were measured. CPM of each sample was counted (*n* = 5 per group). Data are mean ± SEM. One-way ANOVA with Tukey’s multiple comparison test was used to calculate statistical significance. Significance: *P < 0.05.

Metabolic shifts or “switches” play critical roles in the health and disease processes of kidney cells and recent studies suggest that such alterations in fuel preference are associated with mesenchymal transformation in kidney cells (*16*, *47*, *49*, *58*–*60*). Our data show that diabetes suppressed the expression of proteins associated with FAO such as carnitine palmitoyltransferase 1a (CPT1a), peroxisome proliferator-activated receptor gamma coactivator 1-alpha (PGC1α), and sirtuin 3 (SIRT3) in the kidneys from diabetic control mice but not in the kidneys from Angplt4^−/−^ mice (Fig. 2A). Diabetes increased the expression of regulators that control the oxidation of fuel types including pyruvate kinase muscle type 2 (PKM2), pyruvate dehydrogenase kinase 4 (PDK4), hypoxia-inducible factor 1–alpha (HIF1α), and the EMT regulator Snail1 in the kidneys from diabetic control mice whereas these effects were not prominent in kidneys from Angplt4^−/−^ mice (Fig. 2, B and C). These data are aligned with the results of FAO and metabolic flexibility assays in these kidneys. The kidneys from diabetic control mice demonstrated diminished FAO and basal oxygen consumption rates (OCRs) whereas the kidneys from diabetic Angplt4^−/−^ mice demonstrated increased FAO and OCR levels (Fig. 2D and fig. S6C). Increased lipid deposition and TGs and diminished adenosine triphosphate (ATP) levels were observed in the kidneys of diabetic control mice compared to those from nondiabetic controls. Conversely, the kidneys of diabetic Angplt4^−/−^ mice had decreased lipid deposition and TGs and elevated ATP levels compared to those from diabetic control mice (fig. S7, A to C). Fatty acid synthase (*FASN*) mRNA expression was down-regulated in the kidneys of Angplt4^−/−^ mice when compared to those of diabetic littermate controls (fig. S7D). In addition, FAO was significantly suppressed in tubules, endothelial cells, and podocytes in diabetic mice when compared to those cell types in nondiabetic mice. Tubules, endothelial cells, and podocytes from diabetic Angplt4^−/−^ mice had elevated levels of FAO when compared to diabetic control mice, suggesting a critical role of *Angptl4* in the regulation of FAO in diabetic kidneys (Fig. 2E).

### Angptl4 deficiency in podocytes suppresses fibrogenic phenotypes

**Fig. 3.**
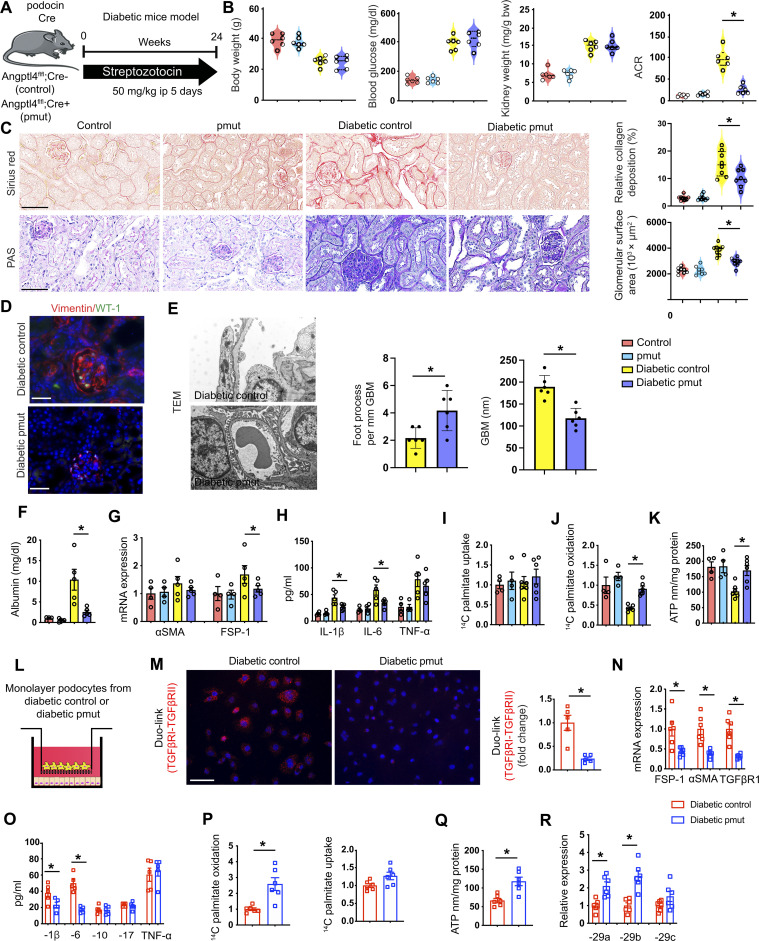
Metabolic reprogramming by podocyte Angptl4 loss protects against DKD. (**A**) Schematic diagram showing induction of diabetes. Art was created using Servier medical art illustrations. (**B**) Physiological parameters. *n* = 6 mice per group combined from two replicate experiments. (**C**) Sirius red and PAS staining. Representative images (original magnification, 300×) are shown. Quantification was performed using ImageJ. Scale bar, 50 μm. (**D**) Immunofluorescence analysis of vimentin/WT-1. FITC-labeled WT-1, rhodamine-labeled vimentin, and DAPI (nuclei, blue) were used. Representative merged (original magnification, 400×) images. Scale bar, 50 μm. *n* = 5 mice per group. (**E**) Representative transmission electron microscopy images. *n* = 3 per group. Scale bar, 1 μm. Relative density of podocyte foot processes and GBM thickness were calculated using ImageJ. (**F**) After 48 hours, albumin was measured in the cell media of cultured isolated podocytes. (**G**) qPCR mRNA gene expression of α*SMA* and *FSP-1* in isolated podocytes. *n* = 4 per nondiabetic group and *n* = 5 per diabetic group. (**H**) IL-1β, IL-6, and TNFα levels measured in culture media. (**I**) Radiolabeled [C^14^]palmitate uptake analysis and (**J**) [^14^C] palmitate oxidation in isolated podocytes. CPM of each sample was counted. (**K**) ATP measurement via calorimetric assay in isolated podocytes. (**L**) Schematic diagram of coculture setup. (**M**) Representative Duolink In Situ images of TGFβR1/2 from tubules that were cocultured with podocytes and corresponding quantification. Original magnification 400×. (**N**) qPCR gene expression analysis of *FSP-1*, α*SMA,* and *TGFβR1.* (**O**) Measurement of IL-1β, IL-6, IL-10, IL-17, and TNFα levels in the cell supernatant. (**P**) Radiolabeled [^14^C]palmitate uptake and oxidation were measured in indicated groups. CPM of each sample was counted. (**Q**) ATP measurement in isolated tubules. (**R**) qPCR gene expression analysis of miR-29a-3p, miR-29b-3p, and miR-29c-3p in tubules cocultured with the indicated podocytes. Data are mean ± SEM. One-way ANOVA with Tukey’s multiple comparison test was used to calculate statistical significance. Significance: *P < 0.05.

Mice with conditional alleles (Angptl4^fl/fl^) were generated by removal of the gene trap cassette with Flp recombinase using the original tm1a mice. To test the role of *Angptl4* in podocytes, we generated podocyte-specific Angptl4 mutant mice (pmut) by crossing Angptl4^fl/fl^ mice with mice carrying the *podocin Cre* driver (Angptl4^fl/fl^; *podocin Cre+*) (Fig. 3A). In Angptl4^fl/fl^ mice, the expression of the LacZ reporter gene is regulated by the ANGPTL4 promoter in the original tm1a mice, and expression of ANGPTL4 can be detected using an antibody against β-galactosidase (β-gal). Therefore, we analyzed β-gal staining in pmut and control mice. Pmut mice have higher expression of β-gal–positive cells in the glomeruli when compared to littermate controls (fig. S8A). Pmut mice had significantly suppressed levels of *Angptl4* expression in isolated podocytes when compared to control mice (fig. S8B). Nondiabetic and diabetic pmut mice did not show any remarkable alterations in body weight, blood glucose, or kidney weight when compared to their nondiabetic and diabetic controls, respectively (Fig. 3B). However, diabetic pmut mice had suppressed albumin-to-creatinine ratios (ACRs), relative fibrosis, glomerulosclerosis, and vimentin levels when compared to diabetic control mice (Fig. 3, B to D, and fig. S8C). αSMA and Collagen I mRNA expression were significantly down-regulated in diabetic pmut mice (fig. S8D). Glomerular ultrastructure was analyzed by transmission electron microscopy and diabetic control mice displayed some podocyte foot process effacement while pmut did not show such a prominent effect (Fig. 3E).

Increased lipid deposition, higher TGs, increased lipid uptake, decreased lipid oxidation, and decreased ATP levels were observed in the kidneys of diabetic control mice when compared to those from nondiabetic controls (fig. S9, A to D). The kidneys from diabetic pmut mice had decreased lipid deposition, decreased TGs, decreased lipid uptake, and increased lipid oxidation and ATP levels compared to the kidneys from diabetic controls (fig. S9, A to D). *FASN* mRNA expression was down-regulated in the kidneys from diabetic pmut as compared to diabetic littermate controls (fig. S9E). There were no remarkable changes in the kidney cholesterol levels between diabetic control and diabetic pmut mice; cholesterol levels were overall increased in the diabetic mice, as expected (fig. S9F).

Podocytes from diabetic pmut mice had suppressed albumin permeability when compared to podocytes from diabetic control mice as measured by albumin assay in cell culture media (Fig. 3F). There was no remarkable alteration in the gene expression of α*SMA* between podocytes from the diabetic pmut when compared to diabetic control podocytes, though *FSP-1* expression was significantly decreased in diabetic pmut podocytes (Fig. 3G). Culture media from diabetic pmut podocytes had significantly suppressed levels of IL-1β and IL-6 compared to media from diabetic control podocytes (Fig. 3H). Podocytes from diabetic pmut mice had no change in lipid uptake; however, lipid oxidation and ATP levels were higher when compared to podocytes from diabetic control mice (Fig. 3, I to K).

Next, podocytes from control mice and pmut mice were cocultured with isolated control tubules (Fig. 3L). Suppressed levels of TGFβR1/TGFβR2 heterodimerization (DuoLink proximity ligation assay); down-regulated mRNA expression of FSP-1, αSMA, and TGFβR1; and mitigated levels of the pro-inflammatory cytokines IL-1β and IL-6 in tubules cultured with podocytes from diabetic pmut mice were observed (Fig. 3, M to O). The tubules cultured with podocytes from pmut mice did not show any change in lipid uptake, but did show higher levels of lipid oxidation, ATP, and miR-29 expression levels (Fig. 3, P to R). These findings suggest that *Angptl4* deficiency in podocytes protects against EMT in renal tubular cells.

Furthermore, we tested the effect of podocyte *Angptl4* deficiency on the mesenchymal activation of endothelial cells. Podocytes from control mice and pmut mice were cocultured with control endothelial cells (fig. S9G). Again, we observed down-regulated mRNA expression of FSP-1, αSMA, and TGFβR1 and suppressed expression of the pro-inflammatory cytokines IL-1β and IL-6 in endothelial cells cultured with podocytes from diabetic pmut mice (fig. S9, H and I). Endothelial cells cultured with podocytes from pmut mice did not show any change in lipid uptake, but again showed higher levels of lipid oxidation and ATP levels (fig. S9, J and K). These cells also showed decreased permeability as measured by the concentration of fluorescein isothiocyanate (FITC)–dextran (fig. S9L). These findings suggest that podocyte *Angptl4* deficiency also protects against EndMT in endothelial cells. To further evaluate the pmut mice in a model of type 2 diabetes, we provided high-fat diet (HFD) to control and pmut mice for 16 weeks and analyzed renal function and structure (fig. S10A). There were no differences in body weight, blood glucose, or kidney weight; however, ACR was significantly lower in pmut mice (fig. S10, B to E). Relative area of fibrosis and relative collagen deposition were lower in HFD-fed pmut mice when compared to HFD-fed control (fig. S10F).

### Angptl4 deficiency in tubules suppresses fibrogenic phenotypes

**Fig. 4.**
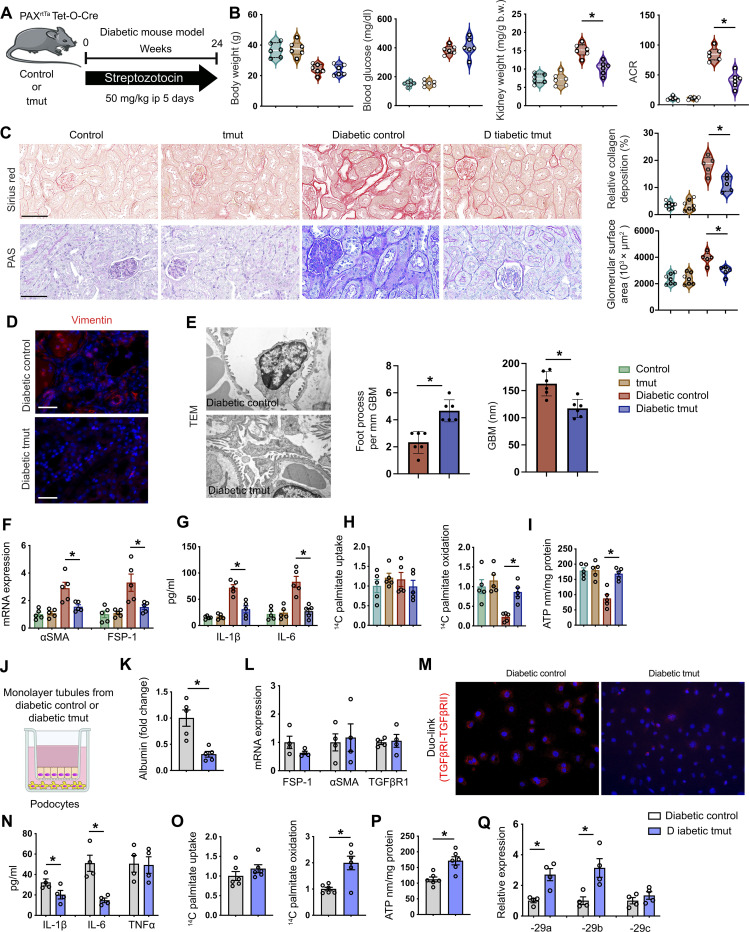
Metabolic reprogramming by tubular Angptl4 loss protects against DKD. (**A**) Schematic diagram showing induction of diabetes. Art was created using Servier medical art illustrations. (**B**) Physiological parameters. *n* = 5 mice per group combined from two replicate experiments. (**C**) Sirius red and PAS staining in kidneys (original magnification, 300×). Quantification was performed using ImageJ. Scale bar, 50 μm. (**D**) Immunofluorescence analysis of vimentin. Rhodamine-labeled vimentin and DAPI (nuclei, blue) were used. Representative merged (original magnification, 400×) images are shown. Scale bar, 50 μm. *n* = 5 mice per group. (**E**) Representative transmission electron microscopy images. *n* = 3 per group. Scale bar, 1 μm. Quantification was calculated using ImageJ software. Six independent images of the staining were analyzed. (**F**) qPCR gene expression of α*SMA* and *FSP-1* in tubules from nondiabetic control and tmut and diabetic control and tmut mice. *n* = 5 per group. (**G**) IL-1β and IL-6 measurement in culture tubules. *n* = 5 per group. (**H**) Radiolabeled [C^14^]palmitate uptake analysis and radiolabeled [^14^C]palmitate oxidation were measured in tubules. CPM of each sample was counted. (**I**) ATP measurement in cultured tubules. (**J**) Schematic diagram showing coculture experimental setup. (**K**) After 48 hours, albumin was measured in the cell media of podocytes cocultured with tubules. (**L**) Gene expression analysis of *FSP-1***,** α*SMA*, and *TGFβR1* in podocytes cocultured with tubules. (**M**) Representative Duolink In Situ images of TGFβR1/2 from podocytes that were cocultured with tubules and corresponding quantification (original magnification, 400×). (**N**) Measurement of IL-1β, IL-6, and TNFα levels in the cell supernatant. (**O**) Radiolabeled [^14^C]palmitate uptake and oxidation. CPM of each sample was counted. (**P**) ATP measurement in podocytes. (**Q**) qPCR gene expression analysis of miR-29a-3p, miR-29b-3p, and miR-29c-3p in podocytes that were cocultured with tubules from diabetic control and diabetic tmut mice. Data are mean ± SEM. One-way ANOVA with Tukey’s multiple comparison test was used to calculate statistical significance. Significance: *P < 0.05.

To further investigate the role of *Angptl4* in tubules, we generated tubule-specific Angtpl4 mutant mice (tmut) by crossing Angptl4^fl/fl^ mice with mice carrying the Tet-inducible *Pax8 Cre* driver (Angptl4^fl/fl^; Tet+; *Pax8 Cre+*) (Fig. 4A). We used the same β-gal staining strategy to evaluate the expression of ANGPTL4 in tmut mice. Tmut mice have higher expression of β-gal–positive cells in the tubules when compared to littermate controls (fig. S11A) and tmut mice had significantly suppressed levels of *Angptl4* expression in isolated tubules when compared to control mice (fig. S11B). Diabetic tmut mice did not show any remarkable change in body weight or blood glucose levels when compared to diabetic controls (Fig. 4B). Diabetic tmut mice had decreased kidney weight, lower ACR, decreased area of fibrosis, less glomerulosclerosis, and suppressed vimentin levels when compared to diabetic controls (Fig. 4, C and D, and fig. S11C). αSMA and Collagen I mRNA expression were significantly down-regulated in diabetic tmut mice when compared to diabetic controls (fig. S11D). Ultrastructural analysis by transmission electron microscopy revealed podocyte foot thickening and effacement in diabetic control mice, which was less pronounced in diabetic tmut mice (Fig. 4E).

Increased lipid deposition, higher TGs, increased lipid uptake, lipid oxidation, and decreased ATP levels were observed in the kidneys of diabetic control mice when compared to nondiabetic controls. In contrast, the kidneys of diabetic tmut mice had diminished lipid deposition, lower TGs, lower lipid uptake, and elevated lipid oxidation and elevated ATP levels compared to diabetic control mice (fig. S12, A to E). *FASN* mRNA expression was down-regulated in kidneys from diabetic tmut compared to diabetic littermate controls (fig. S12F). There were no remarkable changes in the kidney cholesterol levels between diabetic control and diabetic tmut mice (fig. S12G).

Tubules from diabetic tmut mice had suppressed FSP-1 and αSMA gene expression (Fig. 4F). Culture media from tmut tubules mice had significantly suppressed levels of IL-1β and IL-6 (Fig. 4G). Isolated tubules from diabetic tmut mice had no change in lipid uptake but did show higher lipid oxidation and ATP levels when compared to tubules from diabetic control mice (Fig. 4, H and I). Tubules from control mice and tmut mice were then cocultured with control podocytes (Fig. 4J). Podocytes that were cocultured with diabetic tmut mice tubules had suppressed albumin permeability as assayed in the culture media (Fig. 4K) and also did not show any significant change in mRNA expression of FSP-1, αSMA, and TGFβR1. However, these podocytes did show suppressed levels of TGFβR1 and TGFβR2 heterodimerization as assessed by DuoLink assay, and lower levels of IL-1β and IL-6 in culture media (Fig. 4, L to N). There was no remarkable change in lipid uptake; however, these podocytes had higher levels of lipid oxidation and ATP and increased miR-29a and miR-29b expression levels when compared to podocytes cultured with control tubules (Fig. 4, O to Q). These findings suggest that *Angptl4* deficiency in tubules protects against podocyte damage in diabetes.

Furthermore, we tested the effect of tubular *Angptl4* deficiency on mesenchymal activation in endothelial cells. Tubules from control mice and tmut mice were cocultured with control endothelial cells (fig. S12H). We observed lower mRNA expression of FSP-1, αSMA, and TGFβR1 as well as suppressed levels of IL-1β and IL-6 in the media from endothelial cells cultured with tubules from diabetic tmut mice compared to those cultured with control tubules (fig. S12, I and J). Endothelial cells cultured with tubules from tmut mice did not show any change in lipid uptake, but again showed higher levels of FAO and ATP (fig. S12, K and L). Endothelial permeability was suppressed in these cells (fig. S12M). Together, these findings suggest that tubular *Angptl4* deficiency protects against mesenchymal activation in endothelial cells. We then evaluated this tmut mouse in a model of type 2 diabetes by feeding HFD to control and tmut mice for 16 weeks with subsequent analysis of renal function and structure (fig. S13A). There was no remarkable difference in the body weight, blood glucose, and kidney weight; however, ACR was significantly lower in tmut mice (fig. S13, B to E). Relative area of fibrosis and relative collagen deposition were lower in HFD-fed tmut mice when compared to HFD-fed control mice (fig. S13F).

### Podocyte- and tubule-secreted Angptl4 interact with integrin β1 and influence dipeptidyl peptidase-4–integrin β1 interactions

**Fig. 5.**
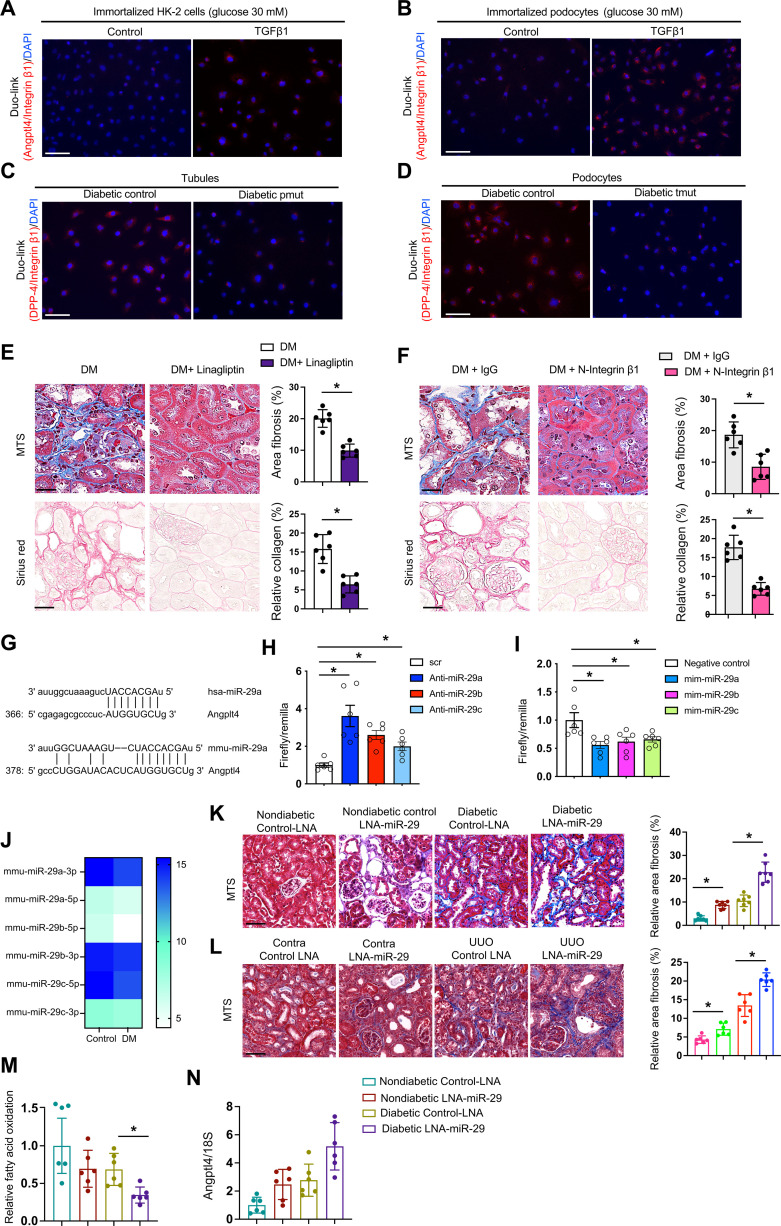
Angplt4 regulates DPP-4/β-integrin interactions. (**A**) Proximity between Angptl4 and β-integrin in HK-2 cells and (**B**) podocytes in cultured HK-2 cells treated with TGFβ1 (10 ng/ml) for 48 hours. Images at 400× original magnification were obtained from six different areas. Scale bar, 50 μm. (**C**) Proximity between DPP-4 and β-integrin in tubules cocultured with podocytes from the indicated mice. Images at 400× original magnification, were obtained from six different areas. Scale bar, 50 μm. (**D**) Proximity between DPP-4 and β-integrin in podocytes cocultured with tubules from the indicated mice. For each slide, images at 400× original magnification were obtained from six different areas. Scale bar, 50 μm. (**E**) Representative MTS and Sirius red staining in kidneys of control and linagliptin-treated diabetic mice. Scale bar, 50 μm. *n* = 6 mice per group. (**F**) Representative MTS and Sirius red staining in kidneys from IgG control-treated and N–Integrin β1–treated diabetic mice. Scale bar, 50 μm. *n* = 6 mice per group. (**G**) Data from www.mirbase.org suggest miR-29a nucleotide sequence alignment with the 3′UTR of Angptl4 mRNA. (**H**) Angptl4 3′UTR transcriptional activity measurement in the presence of antagomirs or (**I**) mimics of microRNA 29a, b, and c. Two independent experiments were analyzed. (**J**) microRNA array analysis of miR-29 family members in control and diabetic mice. (**K**) Representative images of MTS in kidneys after LNA–miR-29 treatment in control and diabetic mice. Scale bar, 50 μm. *n* = 7 mice per group. (**L**) Representative images of MTS in kidneys after LNA–miR-29 treatment in control and unilateral urinary obstruction (UUO)–operated mice. Scale bar, 50 μm. *n* = 5 mice per group. (**M**) Radiolabeled [^14^C]palmitate oxidation and [^14^C]CO_2_ uptake were measured in indicated groups. CPM of each sample was counted. *n* = 6 per group. (**N**) *Angplt4* mRNA expression in the indicated groups. *n* = 6 per group. Data are mean ± SEM. One-way ANOVA was used for the analysis of statistical significance. Significance: *P < 0.05.

Active TGFβ signaling and elevated dipeptidyl peptidase-4 (DPP-4) levels are causative pathways that accelerate renal fibrosis in diabetes (*61*). TGFβ1 stimulates DPP-4–Integrin β1 associated fibrogenesis in diabetic endothelium (*61*). Duo-link In Situ proximity ligation assay demonstrated that TGFβ1 stimulation increased the interaction of Angptl4 with Integrin β1 in high-glucose–treated HK-2 cells and podocytes (Fig. 5, A and B). To investigate the mechanism underlying the interaction between Angptl4 and Integrin β1, we analyzed the protein interaction of DPP-4 and Integrin β1, which is critical for mesenchymal activation signal transduction. Podocytes from diabetic control mice had closer proximity of DPP-4 and Integrin β1 when compared to those from nondiabetic controls, and podocytes from diabetic pmut mice had further proximity between DPP-4 and Integrin β1 when compared to those from the diabetic control mice (fig. S14A). Similar results were found in the cultured tubules from diabetic control and tmut mice (fig. S14A). To test whether Angptl4 interacts with DPP-4 and Integrin β1, we used small interfering RNA (siRNA)–mediated knockdown of DPP-4 and Integrin β1 and performed proximity assays between Angptl4 and both DPP-4 and Integrin β1. TGFβ1 stimulated Angptl4–Integrin β1 interactions. Both DPP-4 knockdown and Integrin β1 knockdown suppressed this interaction in cultured podocytes as well as in HK-2 cells (fig. S14B). Similarly, TGFβ1 stimulates Angptl4–DPP-4 interactions and either DPP-4 knockdown or Integrin β1 knockdown suppressed this interaction in both cultured podocytes and HK-2 cells (fig. S14C). Further, we observed closer proximity between DPP-4 and Integrin β1 in tubules, which were cocultured with diabetic pmut mice podocytes and in podocytes that were cocultured with diabetic tmut tubules when compared to respective diabetic controls, suggesting that either podocyte- or tubule-specific Angptl4 plays a critical role in the DPP-4–Integrin β1 interaction-mediated mesenchymal signal transduction (Fig. 5, C and D). The kidneys of diabetic pmut and diabetic tmut had suppressed protein expression levels of DPP-4, TGFβ1, and phospho-smad3 when compared to respective diabetic controls (fig. S15). To test the Angptl4-associated pathogenic roles of DPP-4 and Integrin β1, we treated diabetic control mice with kidney fibrosis with linagliptin, a DPP-4 inhibitor or neutralizing antibody to Integrin β1. Both linagliptin and Integrin β1 neutralization reversed the fibrotic phenotypes and significantly suppressed *Angptl4* expression in diabetic kidneys (Fig. 5, E and F, and fig. S16, A and B). To investigate why DPP-4 and Angptl4 levels were elevated in diabetes, we performed miRNA array analysis in control and diabetic mice. miR-29 family members have emerged as key players that are associated with targeting DPP-4 mRNA in endothelial cells in diabetes (*61*). Of note, miR-29s have conserved site binding sequences at the 3′ untranslated region (3′UTR) of Angplt4 mRNA (Fig. 5G). Luciferase assay analysis revealed that inhibiting miR-29 using an antagomir elevated the Angptl4 3′UTR luciferase activity while use of a mimic of miR-29 suppressed the luciferase activity of Angptl4 3′UTR (Fig. 5, H and I). microRNA array analysis revealed suppressed expression of miR-29 family members in the kidneys of diabetic mice when compared to those of nondiabetic control mice (Fig. 5J). Diabetic mice subjected to miR-29 inhibition, through administration of LNA–miR-29, had higher kidney weight and albumin-to-creatinine ratios and significantly worsened fibrosis (Fig. 5K and fig. S16C). Suppression of all miR-29s with LNA administration was confirmed by relative gene expression assays (fig. S16D). miR-29 inhibition in UUO-operated mice showed more fibrosis when compared to control-LNA (Fig. 5L). Treatment with LNA–miR-29 suppressed FAO levels (Fig. 5M) and elevated *Angptl4* gene expression levels (Fig. 5N). These data suggest that suppression of miR-29 is a key factor that contributes to higher DPP-4 and Angptl4 levels, which, in turn, interacts with integrin β1 and promotes mesenchymal activation in tubules and podocytes leading to dysfunction.

Clement *et al.* (*43*) found a pro-proteinuric form of Angptl4 in glomeruli, which is hyposialylated. Conversion of Angplt4 from the hyposialylated to the sialylated form by treatment with *N*-acetyl-d-mannosamine suppressed the level of proteinuria and disease phenotype in this mouse model of nephrotic syndrome. Therefore, we tested the sialic acid precursor *N*-acetyl mannosamine (NAM) in our mouse model of DKD. NAM did not cause any differences in body weight, blood glucose, or blood pressure but significantly suppressed albuminuria and kidney weight. NAM treatment significantly suppressed kidney weight/body weight ratio, ACR, fibrosis, collagen deposition, and glomerulosclerosis in diabetic mice (fig. S17).

### Angptl4 deficiency in podocytes and tubules reprograms mitochondrial damage and STING activation in diabetes

**Fig. 6.**
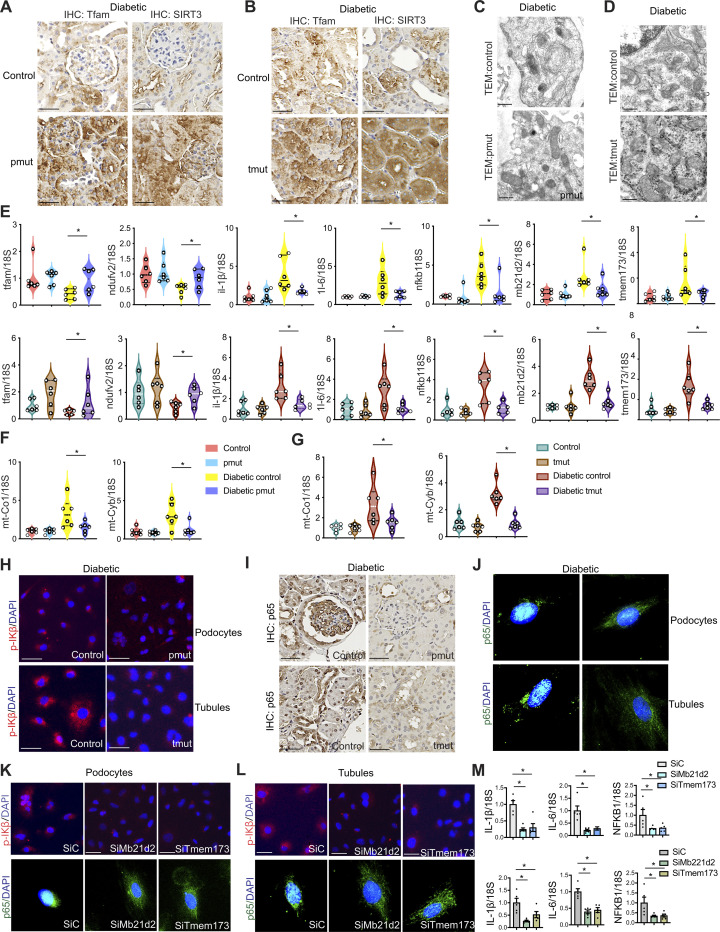
Angptl4 deficiency in podocytes and tubules mitigates mitochondrial damage and STING activation in diabetes. (**A**) Immunohistochemical analysis of mitochondrial proteins TFAM and SIRT3 in the kidneys of diabetic control and pmut mice and (**B**) diabetic control and tmut mice. Representative images are shown. *n* = 6 per group. Scale bar, 50 μm. (**C**) Representative transmission electron microscopy images of mitochondria in the podocytes of diabetic control and pmut mice and (**D**) diabetic control and tmut mice. *n* = 3 per group. (**E**) Relative gene expression levels of *TFAM*, *NDUFV2*, *IL-1*β, *IL-6*, *NFKB1*, *MB21D2,* and *TMEM173* in kidneys of nondiabetic and diabetic control and pmut and tmut mice. *n* = 6 per group. 18*S* was used as an internal control. (**F** and **G**) Relative gene expression levels of *mt-Co1* and *mt-Cyb* in the cytosolic fractions of kidneys from nondiabetic and diabetic pmut and tmut mice. *n* = 6 per group. 18*S* was used as an internal control. (**H**) Immunofluorescence analysis of p-IKB/DAPI in isolated podocytes and tubular cells from diabetic pmut and tmut mice. *n* = 6 per group. p-IKB, rhodamine-labeled, and DAPI (nuclei, blue). Representative images are shown. (**I**) Immunohistochemical analysis of p-65 in kidneys from diabetic pmut and tmut mice. Representative images (original magnification, 400×) are shown. Scale bar, 50 μm. *n* = 6 mice per group. (**J**) Immunofluorescence analysis of p-65/DAPI in isolated podocytes and tubular cells from diabetic pmut and tmut mice. *n* = 5 per group. p-65, FITC-labeled and DAPI (nuclei, blue). Representative images are shown; three independent experiments were analyzed. (**K** and **L**) Immunofluorescence analysis of p-IKB/DAPI and p-65/DAPI in siRNA-Mb21d2 and siRNA-Tmem173 transfected podocytes and tubules. *n* = 5 per group. p-IKB rhodamine-labeled, p-65 FITC-labeled, and DAPI (nuclei, blue). Representative images are shown. (**M**) Relative gene expression levels of *IL-1*β, *IL-6*, and *NFKB1* in siRNA-Mb21d2 and siRNA-Tmem173 transfected podocytes and tubules. *n* = 5 per group. 18*S* was used as an internal control. Data are mean ± SEM. One-way ANOVA was used for the analysis of statistical significance. Significance: *P < 0.05.

Defective lipid metabolism and related lipotoxicity are detrimental not only to the cells but also to cell organelles, including mitochondria (*47*, *62*, *63*). Damaged mitochondria release mitochondrial DNA, which is sensed by the cytosolic cGAS–stimulator of interferon genes (STING) DNA sensing pathway (cGAS-STING pathway), which subsequently up-regulates the transcription of pro-inflammatory genes (*62*). Kidneys from diabetic control mice showed decreased expression of the key mitochondrial proteins TFAM and SIRT3 compared to kidneys from diabetic pmut and tmut mice (Fig. 6, A and B). Transmission electron microscope imaging analysis showed abnormal mitochondrial structures in diabetic control mice whereas pmut mice and tmut kidneys exhibited a more typical mitochondrial ultrastructure (Fig. 6, C and D). Relative mRNA expression levels of *TFAM* and *NDUFV2* were lower in diabetic controls compared to nondiabetic controls, and they were increased in the kidneys of diabetic pmut mice and diabetic tmut mice as compared to their respective diabetic controls (Fig. 6E). Relative expression levels of the pro-inflammatory genes *IL-1*β, *IL-6*, *NFKB1*, cGAS gene *Mb21d2*, and STING gene *TMEM173* were significantly higher in diabetic controls when compared to nondiabetic controls. The expression levels of these same five genes were remarkably suppressed in the kidneys of diabetic pmut and diabetic tmut mice when compared to the respective diabetic controls (Fig. 6E). We did not observe any remarkable difference in the plasma cytokine levels of IL-1β, IL-5, IL-6, CCL3, CCL5, or MoGCSF in diabetic pmut or tmut mice when compared to their controls; however, we observed that renal IL-1β, IL-6, and MCP-1 levels were significantly higher in diabetic controls of both genotypes when compared to nondiabetic mice (fig. S18, A to D), while renal IL-1β, IL-6, and MCP-1 levels were significantly lower in diabetic pmut and diabetic tmut mice when compared to their respective diabetic controls (fig. S18, C and D). Relative gene expression levels of the mitochondrial genes *mt-Co1* and *mt-Cyb* were up-regulated in the cytosolic fractions of diabetic control mice while diabetic pmut mice and tmut mice showed down-regulated expression levels of these genes (Fig. 6, F and G). Immunofluorescent staining revealed that expression of phospho-IKB was higher in isolated podocytes and tubules from diabetic controls and much lower in diabetic pmut and diabetic tmut (Fig. 6H). Similarly, nuclear p-65 expression levels were higher in the kidneys and isolated cells from diabetic controls while relatively suppressed in tissues from diabetic pmut and tmut mice (Fig. 6, I and J). Nondiabetic mice of each genotype showed no differences in expression of either p-IKB or p-65 (fig. S19). Gene silencing of Mb21d2 and TMEM173 resulted in the reduction in the levels of phospho-IKB, and nuclear p-65 expression as well as the gene expression levels of *IL-1*β, *IL-6*, and *NFKB1* in podocytes and tubules (Fig. 6, K to M). These data suggest that Angptl4 deficiency in podocytes and tubules suppresses diabetes-associated cytokines, mtDNA release, and cGAS-STING pathways.

### Kidney-specific Angptl4 ASO improves the fibrogenic phenotype in diabetic mice

We also analyzed the effects of some well-known and effective drugs, such as dichloroacetate (DCA), which causes increased conversion of pyruvate to acetyl-CoA, the glycolysis inhibitor 2-deoxy-glucose (2-DG), the angiotensin-converting enzyme inhibitor (ACEi) imidapril, and the lipid-lowering drugs fenofibrate and simvastatin, on *Angptl4* expression in diabetic kidneys. The anti-inflammatory peptide *N*-acetyl-seryl-aspartyl-lysyl-proline (*Ac-SDKP*) was also used, both alone and in combination with ACEi. DCA, 2-DG, Ac-SDKP, Ac-SDKP + ACEi, ACEi, and fenofibrate significantly suppressed fibrosis and *Angptl4* expression, suggesting that blockade of kidney-specific *Angptl4* could be an important strategy in the management of DKD (fig. S20, A and B).

**Fig. 7.**
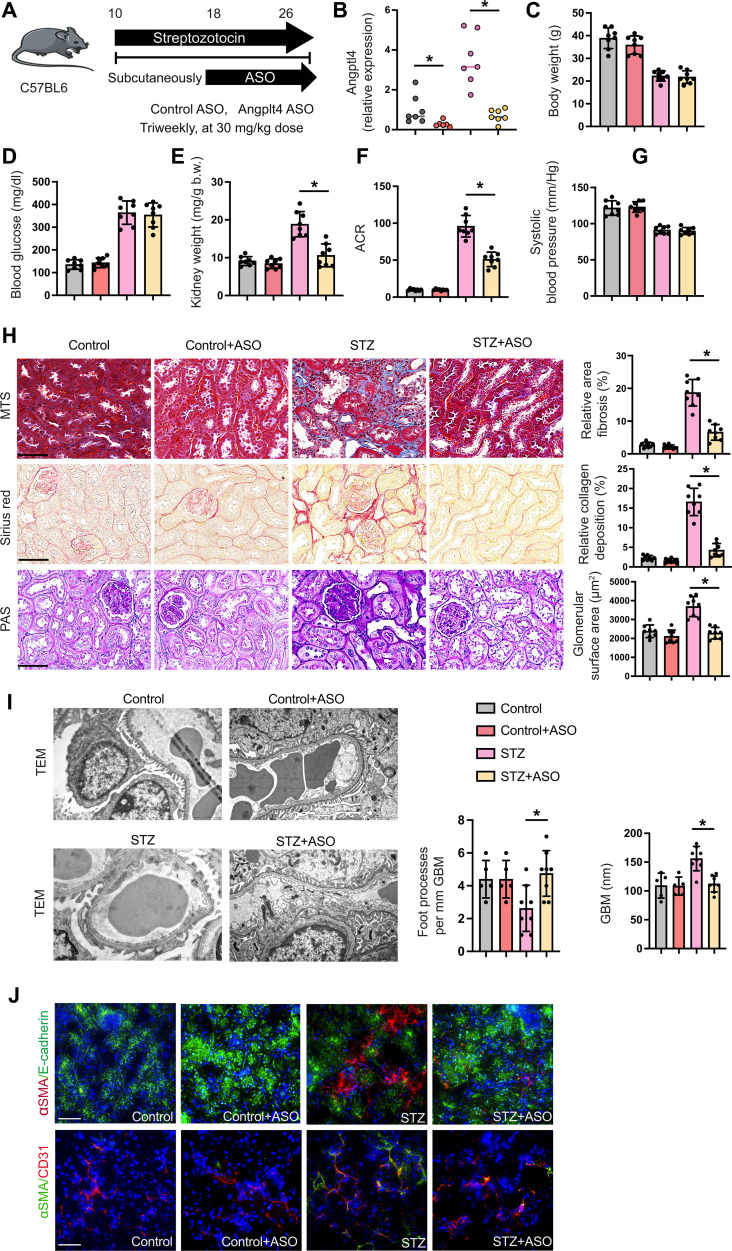
Kidney-specific ASO treatment protects against DKD. (**A**) Schematic diagram showing the treatment protocol of control ASO and kidney-specific Angplt4 ASO treatment in diabetic and nondiabetic C57BL/6 mice. Five doses of STZ (50 mg kg^−1^ day^−1^ ip) were injected to induce diabetes; after 16 weeks, the mice received either control ASO or Angptl4 ASO for 8 weeks. Art was created using Servier medical art illustrations. (**B**) RT-qPCR gene expression analysis of *Angptl4* in kidneys of nondiabetic and diabetic, and control ASO- and Angplt4 ASO-treated mice. *n* = 7 per group. (**C** to **G**) Physiological parameters of nondiabetic and diabetic, and control ASO- and Angplt4 ASO-treated mice. *n* = 8 mice per group, combined from two replicate experiments. (**H**) Representative histologic images from MTS, Sirius red, and PAS staining in kidneys from nondiabetic and diabetic, and control ASO- and Angplt4 ASO-treated mice (original magnification, 300×). Relative area of fibrosis (%) and relative collagen (%) were quantified using ImageJ. Scale bar, 50 μm. (**I**) Representative transmission electron microscopy images are shown. *n* = 3 per group. Scale bar, 1 μm. Relative density of podocyte foot processes and GBM thickness were calculated using ImageJ software. Six independent images of the staining were analyzed. (**J**) Immunofluorescence analysis of αSMA/E-cadherin and αSMA/CD31 in kidneys of ASO treatment in control and diabetic mice. FITC-labeled E-cadherin, rhodamine-labeled αSMA, and DAPI (nuclei, blue), and FITC-labeled αSMA, rhodamine-labeled CD31, and DAPI (nuclei, blue) were used. Representative merged (original magnification, 400×) images are shown. Scale bar, 50 μm. *n* = 8 mice per group. Three independent experiments were analyzed. Data are mean ± SEM. One-way ANOVA was used for the analysis of statistical significance. Significance: *P < 0.05.

Data from human genetic studies have demonstrated that loss-of-function mutations in the *Angptl4* locus are linked to reduced type 2 diabetes and risk of cardiovascular disease (*54*, *64*–*68*). Given the improved kidney phenotype observed in mice lacking *Angptl4* expression in podocytes and tubules, we evaluated targeted inhibition of *Angptl4* expression. We treated diabetic mice with a kidney-specific antisense oligonucleotide (ASO) against Angptl4, which has high affinity for the kidney cortex. Diabetic mice were injected subcutaneously with Angptl4 ASO or control ASO for 8 weeks (Fig. 7A). After treatment, *Angptl4* mRNA was significantly suppressed in both nondiabetic and diabetic mice (Fig. 7B). Angptl4 ASO did not cause any change in body weight, blood glucose, or blood pressure in either group. However, Angptl4 ASO caused significant reduction in the kidney weight and ACR of diabetic mice (Fig. 7, C to G). Angptl4 ASO did not cause remarkable alterations in nondiabetic control mice, but caused significant reduction in fibrosis, collagen deposition, and glomerulosclerosis in diabetic mice, demonstrating that Angptl4 ASO improves the kidney phenotype (Fig. 7H). Electron microscopy revealed partial restoration of normal podocyte structure and GBM caliber in ASO-treated diabetic mice when compared to vehicle-treated diabetic controls (Fig. 7I). Diabetes enhanced the rate of EndMT and EMT in the kidneys when compared to nondiabetic mice as evidenced by higher colocalization of αSMA in E-cadherin–positive and CD31-positive cells. This effect was significantly suppressed in the Angptl4 ASO-treated kidneys, which approximated the ASO-treated nondiabetic kidneys (Fig. 7J).

## DISCUSSION

This study shows the crucial role of podocyte and tubular *Angptl4* in the fibrogenic process of DKD. Our results demonstrate that these cell type–specific *Angptl4* are key fibrogenic molecules that damage mitochondrial structure and function, release mtDNA, and activate cGAS-STING pathways and DPP-4–Integrin β1 signaling. These processes are linked to activation of mesenchymal trans-differentiation processes in diabetic kidneys. Further, these results demonstrate how two components of this disease, fibrosis and mesenchymal metabolic shifts, are linked in the tubules and podocytes. *Angptl4* is expressed in kidneys; however, its roles there have been incompletely investigated (*69*). Human studies have shown a positive correlation between Angptl4 levels and expression of characteristics of diabetic nephropathy (*70*). In cultured glomerular mesangial cells, Angptl4 deficiency inhibits both the inflammatory response and extracellular matrix accumulation (*71*).

*Angptl4* is a known inhibitor of LPL, an enzyme that catalyzes the hydrolysis of TGs into fatty acids, which are used by peripheral tissues and kidney tubules (*69*). Our data demonstrate that *Angptl4* expression is up-regulated, and LPL expression and its associated activities are down-regulated, in fibrotic kidneys during diabetes. However, while chemical inhibition of LPL in diabetic mice caused an elevation in plasma TGs, it did not alter renal fibrosis, suggesting that LPL is not a key molecule in the development of diabetic kidney fibrosis. Analysis of diabetic kidneys from Angptl4 mutant mice shows suppression of renal fibrosis, proteinuria, pro-inflammatory cytokines, and mesenchymal activation in tubules and endothelial cells when compared to diabetic controls. These results suggest that *Angptl4* is associated with fibrosis, proteinuria, inflammation, and mesenchymal activation in diabetic kidneys. It is clear from these data that Angptl4 is one of the catalysts of renal fibrosis in diabetes and leads to disruption of cytokine and chemokine homeostasis by up-regulating TGFβ signaling. These processes alter metabolic homeostasis by predisposing toward defective fatty acid metabolism and associated mesenchymal activation in tubules and endothelial cells.

These observations implicate renal Angptl4 as a mediator and potential therapeutic target for the treatment of DKD. However, a clear role for Angplt4 in regulating whole-body lipid and glucose metabolism has not been fully elucidated due to lack of availability of relevant tissue-specific knockout mouse models. Severe systemic metabolic complications such as gut inflammation and ascites have limited investigators’ ability to analyze the beneficial functions of Angptl4 deficiency in diabetes (*56*). To capitalize on the specificity of Angptl4 expression in the kidney, we generated podocyte-specific and tubule-specific mutant mice. Clement and colleagues demonstrated the critical role of podocyte-specific Angptl4 in causing proteinuria in nephrotic syndrome (*43*). Angplt4 was found to express in two forms, the sialylated (normal) form and the hyposialylated (abnormal) form. The hyposialylated form is causative for proteinuria. Conversion of the hyposialylated to the sialylated form by treatment with *N*-acetyl-d-mannosamine was noted to significantly suppress the levels of proteinuria in a mouse model of nephrotic syndrome (*43*). Clement *et al.* (*44*) also demonstrated that circulatory sialylated Angplt4 suppresses proteinuria while increasing hypertriglyceridemia in mice. In line with previous findings, our data demonstrate that *N*-acetyl-d-mannosamine treatment in diabetic mice suppressed fibrosis and proteinuria and restored kidney structure in diabetic mice. Further, our data show that blocking Angptl4 in podocytes or tubules effectively suppresses proteinuria, glomerular fibrosis, and interstitial fibrosis in diabetic mice. One limitation of our study is that we could not demonstrate whether Angptl4 in diabetic kidneys exists in the hyposialylated or sialylated form due to the lack of a suitable mouse Angplt4 antibody, thus precluding us from evaluating Angptl4 at the protein level. However, on the basis of previous and current data, we suspect that diabetes may cause renal secretion of the hyposialylated form of Angptl4 protein.

To investigate how Angptl4 accelerates mesenchymal activation, we analyzed lipid levels and FAO in tubular epithelial cells (TECs), endothelial cells, and podocytes. It is evident from our results that, in diabetic kidneys, Angptl4 has a prominent effect on de novo lipogenesis (DNL). Here, we show that elevated TG levels (lipid load) due to defects in lipid oxidation in mitochondria can be mitigated by loss of Angptl4, which reduced lipid load, restored mitochondrial structure and function, and restored FAO in diabetes. Increased FAO as a result of overexpressing Cpt1a improves mitochondrial homeostasis and fibrosis in mice (*72*). Lipid accumulation and expression of genes related to DNL have been shown to be elevated in the kidneys of patients with fibrosis (*73*). Acyl-CoA synthetase short-chain family 2 regulates DNL, causing NADPH depletion and increasing ROS levels, ultimately leading to NLRP3-dependent pyroptosis in kidney tubules (*73*). Dysregulated lipid accumulation leads to mitochondrial damage and defects in FAO (*73*, *74*); these are the key processes in the induction of myofibroblast activation and related fibrogenesis in diabetic kidneys (*47*, *73*). Kidney tubules are enriched in mitochondria due to their high ATP demand and therefore are highly dependent on FAO for ATP, because oxidation of fatty acid generates far more ATP than oxidation of glucose (*74*). Our data suggest that up-regulation of *Angptl4* expression in the kidneys of diabetic mice is associated with increased fibrosis and lipid accumulation, and suppression of FAO. *Angptl4* deficiency resulted in increased FAO, decreased abnormal glucose metabolism, and diminished lipid accumulation, suggesting that metabolic reprogramming and cytokine reprogramming by *Angplt4* deficiency is a crucial step in the antimesenchymal and antifibrotic mechanisms occurring in kidney tubules during diabetes.

Defective lipid and glucose metabolism and related mitochondrial defects are critical in kidney disease development. These abnormal metabolic processes contribute to cellular energy deficits, compromised organ function, and/or lack of proper mitochondrial integrity, which are, in turn, associated with release of mtDNA content into the cytosol, where it is recognized as “foreign” DNA. This recognition inappropriately activates the immune system, triggering pathological inflammation by activating the cytosolic cGAS-STING DNA sensing pathway, resulting in aberrant cytokine expression and immune cell recruitment (*62*, *75*). Blocking this foreign DNA sensor pathway mitigates kidney disease development in diabetic mice and offers an unexplored therapeutic pathway for patients with chronic kidney disease (*62*, *76*). To determine how Angptl4 deficiency suppresses inflammation, we analyzed mtDNA release, which was increased in diabetic tubules and diabetic podocytes from control mice and suppressed in cell-specific Angptl4 knockout mice. Decreased mtDNA release as well as decreased STING activation down-regulated pro-inflammatory cytokine expression in diabetes, suggesting that Angptl4 deficiency can restore mitochondrial homeostasis.

To begin to understand how Angptl4 regulates FAO and defective glycolysis, we analyzed DPP-4–Integrin β1 signaling levels and the associated TGFβ dimerization, which is the first step in activating the TGFβ signaling cascade. Activated TGFβ signaling is one mechanism that can suppress FAO and induce abnormal glycolysis, thereby promoting “mesenchymal metabolic shifts” and related fibrogenesis in renal tubules (*47*, *48*). DPP-4 is a well-known molecule influencing TGFβ-associated EndMT and fibrosis in endothelial cells (*61*, *77*, *78*). Proximity ligation assays revealed that TGFβ1-associated fibrogenesis is linked to closer Integrin β1 and Angptl4 proximity, suggesting that Angptl4 interacts with Integrin β1 and influences activation of the mesenchymal signal cascade in tubules. Podocyte-secreted Angptl4 and tubule-secreted Angptl4 influence the DPP-4–Integrin β1 interaction and TGFβR1 and TGFβR2 dimerization, resulting in a cascade of signaling processes mediated through Smad proteins, primarily Smad3. Phosphorylated Smad3 alters the cytoplasmic pathway and also enters the nucleus where it represses the transcription of FAO genes and induces the transcription of genes related to fibrogenesis in tubules (*47*). In podocytes, augmentation of the DPP-4–Integrin β1 signaling cascade caused loss of GBM, alterations in podocyte cell permeability, changes in foot process effacement, and podocyte cell death. These data indicate that podocyte- and tubule-Angptl4 interact with Integrin β1 and promote DPP-4–Integrin β1 interaction and TGFβ signaling, and that these cumulative effects lead to associated activation of mesenchymal signal transduction in tubules and endothelial cells, foot process effacement, increased albuminuria, and podocyte death.

To further investigate the interaction between DPP-4–Integrin β1 and Angptl4, we analyzed microRNA array data from nondiabetic mice and diabetic mice with severe renal fibrosis. Data suggest miR-29-3p is critical in regulating *Angptl4* expression levels and targets the 3′UTR of its mRNA. miR-29-3p targets the 3′UTR of DPP-4 mRNA in endothelial cells and regulates EndMT and fibrogenesis (*61*). From our data, miR-29b-3p deficiency in TGFβ-treated high-glucose–stimulated tubules and in diabetic tubules is identified as a key regulator of DPP-4 and Angptl4 levels. miR-29b-3p targets DPP-4 and Angptl4 levels in the tubules. Inhibition of miR-29 through LNA–miR-29 treatment has been shown to promote favorable plaque remodeling in atherosclerotic mice (*79*), worsen the features of fibrosis, distort kidney structure, and cause severe proteinuria in mouse models of DKD; in separate studies, miR-29b was reported to be reno-protective in *db/db* mice and STZ-induced diabetic mice (*80*, *81*). These observations suggest that miR-29-3p is a crucial regulator of both *DPP-4* and *Angptl4* mRNA and influences fibrogenesis in diabetic kidneys.

Past studies have shown Angptl4 to be a potential metabolic regulator that is linked to several metabolic diseases (*82*–*84*). In one study, Dewey *et al.* (*64*) used a neutralizing antibody to Angptl4 in both humanized mice and nonhuman primates that resulted in severe side effects and systemic metabolic abnormalities. Our data using whole-body Angptl4 mutant mice and podocyte-specific and tubule-specific mutant mice suggest that Angptl4 has promising drug targetability against DKD. Hence, we evaluated a kidney-specific ASO in our model of DKD. This design is advantageous in that it avoids the unfavorable systemic effects of whole-body Angptl4 loss. The kidney-specific ASO was designed to have the highest affinity for the cortex and limited affinity for the medulla. Our results demonstrate that Angptl4 ASO-treated mice have partially restored kidney structure, substantially suppressed glomerular and cortical fibrosis and improved proteinuria, compared to control ASO-treated mice, without affecting body weight, blood glucose, or blood pressure.

Podocyte- and tubule-Angpl4 are key fibrogenic molecules, and their loss is protective against DKD and fibrogenesis by metabolic reprogramming, which is driven by suppression in DPP-4–Integrin β1 signaling and TGFβ signaling. Kidney-specific Angplt4 ASO reversed the fibrogenic phenotypes of DKD. Together, these data add substantial information to the understanding of Angplt4 biology and offer an exciting possibility as a previously unidentified therapeutic option for the treatment of this condition.

## MATERIALS AND METHODS

### Reagents and antibodies

Rabbit polyclonal antibody against ANGPTL4 (no. 40-9800) was from Invitrogen, and mouse monoclonal anti-αSMA (catalog no. A5228) and mouse monoclonal anti–β-actin (AC-74) (A2228) antibodies were from Sigma (St. Louis, MO). Anti-TGFβR1 (catalog no. ab31013), PPARα (catalog no. ab215270), rabbit polyclonal anti-TGFβRII (catalog no. ab61213), mouse monoclonal anti-vimentin (RV202) (catalog no. ab8978), rabbit polyclonal anti-αSMA (ACTA2) (catalog no. ab5694), anti-HIF1α (catalog no. ab516008) and goat polyclonal anti-Snail1 (catalog no. ab53519) antibodies were purchased from Abcam (Cambridge, UK). Mouse anti–β-catenin antibody (catalog no. 610154) was purchased from BD Biosciences. CPT1a (catalog no. 12252) and rabbit polyclonal anti–E-cadherin antibody (24E10) (catalog no. 3195) antibodies were purchased from Cell Signaling Technology (Danvers, MA, USA). Anti-HSP90 was purchased from BD Biosciences (catalog no. 610419). Rabbit polyclonal anti-smad3 (no. 9513) antibody was obtained from Cell Signaling Technology (Danvers, MA, USA). Rabbit polyclonal anti–phospho-Smad3 (s423 and s425) (600-401-919) antibody was purchased from Rockland Immunochemicals (Gilbertsville, PA, USA) Fluorescence-, Alexa Fluor 647–, and rhodamine-conjugated secondary antibodies were obtained from Jackson ImmunoResearch (West Grove, PA). *TGF*β*1*, *TGF*β*2*, and *TGF*β neutralizing antibodies were purchased from PeproTech (Rocky Hill, NJ).

### Animal experimentation

All experiments were performed according to a protocol approved by the Institutional Animal Care and Use Committee at Yale University School of Medicine and Kanazawa Medical University Japan. The experiments at Yale University were in accordance with the National Institutes of Health (NIH) *Guidelines for the Care and Use of Laboratory Animals* and experiments at Kanazawa Medical University were carried out in accordance with Kanazawa Medical University animal protocols (no. 2014-89, no. 2013-114, and no. 2014-101) and approved by the Institutional Animal Care and Use Committee (no. 2021-20179). For the cell-specific loss of function of Angptl4, we bred the Angptl4 flox/flox mice with podocin-cre mice (pmut) and Pax8^rtTa^-Tet-O-Cre mice (tmut) to generate mice with deletion of Angptl4 in podocytes and tubules, respectively. All mice were on the C57BL/6 background. Six-week-old tmut mice were treated with doxycycline (2 mg/ml + 3% sucrose in drinking water) for 2 weeks before experiments. Some podocin-cre mice were bred to the Terminator mice for the isolation of purified podocytes (*85*), an approach that results in diphtheria toxin receptor expression in all non–Cre-expressing cell types, making these cells susceptible to diphtheria toxin. After this breeding strategy, diphtheria toxin treatment killed non–Cre-expressing cells but spared podocytes, enriching the primary cultures to more than 99% purity.

Induction of diabetes in CD-1 mice and C57BL/6 mice was performed according to previously established experimental protocols (*16*, *86*, *87*). A single intraperitoneal dose of STZ (200 mg/kg) was used to induce diabetes in CD-1 mice. In mice on the C57BL/6 background, diabetes was induced in 10-week-old male mice with five consecutive intraperitoneal doses of STZ (50 mg/kg) in 10 mM citrate buffer (pH 4.5). DPP-4 inhibitor (linagliptin) was provided to 16-week STZ-treated diabetic mice using a dose of 5 mg/kg in drinking water for 8 weeks (*61*). In other experiments, control immunoglobulin G (IgG) and N–Integrin β1 IgG were injected intraperitoneally to 16-week STZ-treated diabetic mice using a dose of 1 mg/kg for 8 weeks.

In one experiment, male mice were randomized to one of four groups 16 weeks after induction of diabetes: (i) untreated, (ii) ACEi (imidapril, 5 mg/kg in drinking water), (iii) DCA (1 g/liter in drinking water), and (iv) 2-deoxyglucose, 500 μg/kg via intraperitoneal injection twice per week. Mice were treated for 8 weeks and compared to untreated diabetic mice. In another set of experiments, male diabetic mice were randomized to one of three groups: (i) untreated, (ii) fenofibrate (100 mg/kg by oral gavage), and (iii) simvastatin (40 mg/kg by oral gavage). All mice had free access to food and water during experiments. Blood was obtained by retro-orbital puncture. Blood glucose concentration was measured using glucose strips. Urine albumin levels were assayed using a Mouse Albumin ELISA Kit (Exocell, Philadelphia, PA). Tissue and blood were harvested at the time of sacrifice. Some kidneys were minced and stored at −80°C for gene expression and protein analysis. Other kidneys were placed immediately in optimal cutting temperature (OCT) compound for frozen sections or 4% paraformaldehyde for histologic staining.

In other experiments, pmut mice and tmut mice along with their respective controls were maintained on HFD containing 40% fat (Research Diets, D12108) for 16 weeks. Tissue and blood were harvested at the time of sacrifice. Some kidneys were minced and stored at −80°C for gene expression and protein analysis. Other kidneys were placed immediately in OCT compound for frozen sections or 4% paraformaldehyde for histologic staining. Before sacrifice, mice were weighed, and blood glucose levels were measured using glucose strips. Spot urine samples for albumin and creatinine levels were collected.

### Kidney-specific ASO treatment

For kidney-specific Angptl4 ASO studies, mice at 10 weeks of age were injected subcutaneously with control ASO or Angptl4 ASO at a dose of 10 or 30 mg kg^−1^ week^−1^ for 8 weeks. Uniform chimeric 16-mer phosphonothioate oligonucleotides containing 2′,4′-constrained ethyl-d-ribose (cEt) groups at positions 1 to 3 and 14 to 16 targeted to murine Angptl4 and a control ASO were synthesized and purified by Ionis Pharmaceuticals as previously described (*88*). The ASO sequences were as follows: *Angptl4* ASO: 5′-AGCTGTAGCAGCCCGT-3′ and control ASO: 5′-ACGATAACGGTCAGTA-3′, with the underlined sequences indicating the cEt-modified bases. ASOs were dissolved in phosphate-buffered saline (PBS) for experiments.

### Blood pressure measurement

Blood pressure measurements were taken using the tail cuff method according to the manufacturer’s instructions. Briefly, mice were trained for 5 days before measurement of blood pressure. After mice were placed on the restraint platform, which was maintained at 33° to 34°C, the tail was placed through the optical sensor and the cuff was compressed. Data are presented as the average of 10 blood pressure measurement cycles.

### Morphological evaluation

The glomerular surface area was measured in 10 glomeruli per mouse using ImageJ software. We analyzed PAS-stained glomeruli from each mouse using a digital microscope screen. Masson’s trichrome-stained images were evaluated by ImageJ software, and the fibrotic areas were estimated (*10*).

### Sirius red staining

Deparaffinized sections were incubated with picrosirius red solution for 1 hour at room temperature. The slides were washed twice with acetic acid solution for 30 s per wash. The slides were then dehydrated in absolute alcohol three times, cleared in xylene, and mounted with a synthetic resin. Sirius red staining was analyzed using ImageJ software, and fibrotic areas were quantified.

### Immunohistochemistry

Paraffin-embedded kidney sections (5 μm thick) were deparaffinized and rehydrated (2 min in xylene, four times; 1 min in 100% ethanol, twice; 1 min in 95% ethanol; 45 s in 70% ethanol; and 1 min in distilled water), and the antigen was retrieved in a 10 mM citrate buffer (pH 6) at 98°C for 60 min. To block endogenous peroxidase, all sections were incubated in 0.3% hydrogen peroxide for 10 min. Immunohistochemistry was performed using the Vectastain ABC Kit (Vector Laboratories, Burlingame, CA and Abcam ab64238). Rabbit polyclonal CPT1a (Abnova; H00001374-DO1P; 1:100) antibody was purchased from Abnova, USA. Rabbit polyclonal PKM2 (Cell Signaling Technology catalog no. 4053, RRID:AB_1904096; 1:100), PDK4 (Abcam catalog no. ab71240, RRID:AB_1269709; 1:100), PGC1α (Cell Signaling Technology catalog no. 2178, RRID:AB_823600; 1:100), and CPT1a (Cell Signaling Technology catalog no. 12252, RRID:AB_2797857; 1:100) antibodies were purchased from Cell Signaling Technology. Goat polyclonal anti-SIRT3 antibody (Santa Cruz Biotechnology catalog no. sc-365175, RRID: AB_10710522; 1:200) was purchased from Santa Cruz Biotechnology. A goat polyclonal anti-Snail antibody (Abcam catalog no. ab53519, RRID: AB_881666; 1:100) was purchased from Abcam (Cambridge, MA, USA). In negative controls, the primary antibody was omitted and replaced with the blocking solution.

### Immunofluorescence

Frozen kidney sections (5 μm) were used for immunofluorescence; double-positive labeling with CD31/αSMA and E-cadherin/αSMA was measured. Briefly, frozen sections were dried and placed in acetone:methanol (1:1) solution for 10 min at −30°C. Once the sections were dried, they were washed twice in PBS for 5 min and then blocked in 2% bovine serum albumin/PBS for 30 min at room temperature. Then, sections were incubated in primary antibody (1:100) for 1 hour and washed in PBS (5 min) three times. Next, the sections were incubated with secondary antibodies for 30 min, washed with PBS three times (5 min each), and mounted with mounting medium with DAPI (4′,6-diamidino-2-phenylindole) (Vector Laboratories, Burlingame, CA). The stained sections were analyzed by fluorescence microscopy. For each mouse, the original magnification of 400× was obtained from six different areas and quantified.

### Multiplex staining

Multiplex staining was analyzed according to the manufacturer’s instructions by using an Opal in situ kit (Waltham, MA, USA). Deparaffinized sections were labeled with E-cadherin [Opal 520 (TSA-FITC)] and Vimentin [Opal 670 (TSA-Cy5)] antibodies for EMT transition analysis while α-SMA [Opal 520 (TSA-FITC)] and CD31 [Opal 670 (TSA-Cy5)] antibodies were used for EndMT analysis. The cell nuclei were labeled with DAPI. In negative controls, the primary antibody was omitted and replaced with blocking solution.

### Proximity ligation Duolink in situ assay

Duolink In Situ kits were used to detect the proximity of DPP-4/Integrin β1 and TGFβR1/R2, as previously described (*78*, *89*). Briefly, cells were passaged into eight-well culture slides (BD Biosciences) in growth medium. The cells were washed with PBS, fixed with 4% paraformaldehyde, and permeabilized with 0.2% Triton X-100. Then, blocking solution was used for 30 min at 37°C, after which the cells were incubated in primary antibody [goat anti–Integrin β1 (1:100) and rabbit anti-Angptl4 (1:100), or rabbit anti-TGFβR1 (1:500) and goat anti-TGFβR2 (1:500), or goat polyclonal DPP-4 (1:100) and rabbit anti–Integrin β1 (1:100)] at 4°C overnight. Cells were treated with two proximity ligation assay probes for 1 hour at 37°C, ligation-ligase solution was added for 30 min at 37°C, and amplification-polymerase solution was added for 100 min at 37°C. The slides were then mounted with DAPI and analyzed by fluorescence microscopy. For each slide, original magnification of 400× was obtained from 6 different areas and quantified.

### Oil red O staining

Briefly, 8-μm kidney sections were dried at room temperature for 15 min and fixed in prechilled acetone solution for 10 min. Slides were washed three times with PBS for 5 min and lastly rinsed in 60% isopropanol for 5 min. Lipids were stained by incubating slides in fresh Oil Red O working solution for 60 min at room temperature and rinsed in 60% isopropanol for 5 s. Slides were washed three times with PBS and 5 min with water and counterstained with hematoxylin. Last, slides were washed in 70% ethanol and mounted with mounting media, and immediately imaged.

### X gal staining

Kidneys were stained with X-gal (Sigma-Aldrich) according to the manufacturer’s instructions.

### EndMT and EMT detection

Frozen sections (5 μm) were used for the detection of EndMT and EMT. Cells undergoing EndMT were detected by double-positive labeling for CD31 and αSMA. Cells undergoing EMT were detected by double-positive labeling for E-cadherin and αSMA. Sections were analyzed and quantified by fluorescence microscopy.

### Transmission electron microscopy

For electron microscopy studies, mice were anesthetized and perfused with 4% paraformaldehyde and kidneys were isolated. Kidney samples (1 mm^3^) were fixed with cacodylic acid buffer containing 1 M cacodylic acid and 25% glutaraldehyde in PBS. After Epon-embedding, an RMC/MTX ultramicrotome (Elexience) was used to cut the tissues into ultrathin sections (60 to 80 nm). Sections were mounted and imaged using a Nikon TE 2000U electron microscope on copper grids and stained with lead citrate and 8% uranyl acetate. A Jeol 1200 EX transmission electron microscope (Jeol LTD) equipped with a MegaView II high-resolution transmission electron microscopy camera was used to observe the copper grids. Electron microscopy was performed by the Cellular and Molecular Physiology Core at Yale University. ImageJ was used for quantitative analysis of electron micrographs. Foot processes were measured from at least 50 μm of GBM for each mouse. Podocyte foot processes and GBM thickness were analyzed by ImageJ. Images were blinded by assigning integer numbers before evaluation by someone other than the scorer.

### Isolation of endothelial cells

Endothelial cells from the kidneys of nondiabetic and diabetic mice were isolated using CD31 magnetic beads. Briefly, kidneys were isolated and minced into small pieces. Using a series of enzymatic reactions by treating the tissue with trypsin and Collagenase type I solution, a single-cell suspension was created. The pellet was dissolved with CD31 magnetic beads, and the CD31-labeled cells were separated with a magnetic separator. The cells were further purified on a column. Cell number was counted by a hemocytometer and cells were plated on 0.1% gelatin-coated petri dishes. Cell purity was measured by flow cytometry (BD FACSDiva) using PE-conjugated CD31 (BDB553373) and FITC-conjugated CD45 (BDB553079), both from Becton Dickinson (USA) (*33*).

### Isolation of kidney TECs

After sacrifice, kidneys from diabetic Angptl4^−/−^ and control littermates were excised and perfused with 4% paraformaldehyde (10 ml) followed by collagenase type 2 digestion (2 mg/ml). After digestion, the cortical regions of the kidneys were used for further processing. The cortical region was minced and digested in collagenase buffer for an additional 5 min at 37°C with rotation to release cells. Digested tissue and cell suspension were passed through a 70-μm cell strainer, centrifuged at 50*g* for 5 min, and washed in PBS for two rounds to collect TECs. Isolated TECs were seeded onto collagen-coated petri dishes and cultured in renal epithelial cell medium (C-26130, PromoCell) supplemented with growth factors for TEC growth (*33*).

### Isolation of primary podocytes

Podocyte isolation was performed as previously described (*34*). Briefly, kidneys were dissected, minced, and digested for 45 to 60 min in a solution of collagenase A (1 mg/ml) (Roche) and DNase I. The resulting suspension was strained through a 100-μm strainer and washed three times with Hanks’ balanced salt solution (HBSS) buffer. Then, the suspension was resuspended in 30 ml of 45% Percoll solution (GE Healthcare BioSciences) in isotonic buffer and centrifuged at 10,000*g* for 60 min at 4°C. Glomeruli were enriched in the top band after centrifugation, and this band was collected. Cells were washed three times with HBSS to remove the Percoll solution. The pellet was resuspended and plated on collagen type I–coated dishes in RPMI 1640 with 9% fetal bovine serum (FBS), penicillin (100 U/ml), streptomycin (100 μg/ml), 100 mM Hepes, 1 mM sodium bicarbonate, and 1 mM sodium pyruvate (*34*).

### Seahorse flux analysis of kidneys

Basal OCRs in kidneys from nondiabetic and diabetic wild-type and Angptl4^−/−^ mice were characterized using the Seahorse Flux Analyzer (Agilent) according to the manufacturer’s instructions. Briefly, mice were fasted overnight, euthanized, and perfused with 1× PBS. Whole kidneys were rapidly dissected, washed in 1× KHB buffer and cut into ~2-mg pieces. Following dissection, kidney pieces were snapped into the wells of an XF Islet Capture Microplate containing 500 ml of XF24 Assay Media [Dulbecco’s modified Eagle’s medium (DMEM) base media containing 1 mM pyruvate, 2 mM glutamine, 5.5 mM glucose, and 100 mM palmitate, pH 7.4]. Kidneys were equilibrated at 37°C for 1 hour in a non-CO_2_ incubator and then assayed on a Seahorse XFe24 Analyzer (Agilent) following a 12-min equilibration period. Respiration rates were measured three times using an instrument protocol of 3-min mix, 2-min wait, and 3-min measure. Flux rates were normalized to tissue weight. Experiments were repeated in three mice per condition.

### TG measurement

Renal TGs were measured using a Triglyceride Calorimetric Assay kit (no. 10010303, Cayman). Approximately 50 mg of kidney tissue was homogenized in NP-40 substitute assay buffer containing protease and phosphatase inhibitors. Homogenates were collected after centrifugation at 10,000*g* for 10 min at 4°C.

### Cholesterol measurement

Renal cholesterol was quantitatively measured using established methods (*73*). Approximately 20 mg of kidney tissue was homogenized in 300 μl of chloroform:isopropanol:NP-40 (7:11:0.1) in a microcentrifuge. The homogenate was centrifuged at 15,000*g* for 10 min, and the liquid layer was collected in a separate microtube. The supernatant was air dried at 50°C to remove chloroform and the samples kept under vacuum pressure for 30 min to remove trace organic solvent. The dried lipids were dissolved in 200 μl of assay buffer and cholesterol measurements were performed as per the manufacturer’s protocol using a kit (#K603-100, Biovision).

### ATP measurement

ATP content was determined using the ATP Colorimetric Assay kit (Biovision), following the manufacturer’s instructions.

### mRNA array analysis

Total RNA was isolated using RNeasy Mini Kit (QIAGEN, Hilden, Germany). The RNA concentration was analyzed at 260/280 nm by photometry. The sense cDNA was prepared using a kit from Ambion (Austin, TX) and target hybridizations were analyzed using a Mouse Gene 1.0 ST Array (Affymetrix, Santa Clara, CA). Hybridization was performed for 17 hours at 45°C in a GeneChip Hybridization Oven 640 (Affymetrix). After washing and staining in a GeneChip Fluidics Station 450, hybridized cDNAs were detected using the GeneChip Scanner 3000. The digitalized image data were processed using the GeneChip Operating Software version 1.4. After hierarchical clustering, the results were presented as a heatmap. The PANTHER biological classification system was used to select specific function-related genes, pathway analysis, and molecular function of selected genes.

### mRNA isolation and qPCR

Total RNA was isolated using standard TRIzol protocol. RNA was reverse transcribed using the iScript cDNA Synthesis kit (Bio-Rad) and qPCR was performed on a Bio-Rad C1000 Touch thermal cycler using the resultant cDNA, as well as qPCR Master mix and gene-specific primers. The list of mouse primers used is presented in table S1 and human primers are shown in table S2. Results were quantified using the delta-delta-cycle threshold (Ct) method (ΔΔCt). All experiments were performed in triplicate and 18*S* was used as an internal control.

### RNA extraction and microRNA array analysis

Frozen kidney tissues were first placed in RNA Later (Life Technologies) for 16 hours at −20°C before subsequent homogenization to avoid RNA degradation while extracting high-quality microRNA.Total RNA was isolated using the miRNeasy Kit (Qiagen, Hilden, Germany). The RNA was quantified with a nanodrop spectrophotometer (ND-1000, Nanodrop Technologies, Wilmington, DE, USA). Ratios of OD_260_/OD_280_ were between 1.9 and 2.0. The integrity of RNA samples was determined using a Bioanalyzer 2100 (Agilent, Santa Clara, CA, USA) and all samples gave RIN values in the range of 8.0 to 8.5. The input for the Agilent microRNA labeling system was 100 ng of total RNA. Dephosphorylated and denatured total RNA was labeled with cyanine 3-pCp and subsequently hybridized to the Agilent Mouse microRNA Microarray Release 15.0 using the microRNA Complete Labeling and Hyb Kit (Agilent Technologies Inc., Santa Clara, CA). Following hybridization for 20 hours, the slides were washed with the Gene Expression Wash Buffer Kit (Agilent, Santa Clara, CA, USA) and measured using an Agilent Scanner G2565BA. Agilent Feature Extraction Software 9.5.1 and GeneSpring GX software 12.5 (Agilent) were used for data processing, analysis, and monitoring. Predicted targets, as extracted from www.mirbase.org, were used to identify the potential putative targets of microRNAs (*90*) for cellular pathways, and annotation enrichments were identified using PANTHER databases. These databases categorize a set of genes from the input gene lists based on annotation similarity and then map them as significantly overrepresented in a biological pathway, thus suggesting that the enriched pathways might play a role in the physiological condition being considered.

### microRNA RNA isolation and qPCR

Complementary DNA was synthesized by a miScript II RT kit (Qiagen) using the hiSpec buffer method. microRNA expression was quantified using the miScript SYBR Green PCR Kit (Qiagen) using 3 ng of complementary DNA. The primers to quantify Mm_miR-29a, Mm_miR-29b, and Mm_miR-29c were the miScript primer assays predesigned by Qiagen. The mature microRNA sequences were 5′UAGCACCAUCUGAAAUCGGUUA for Mm_miR-29a, 5′UAGCACCAUUUGAAAUCAGUGUU for Mm_miR-29b, and 5′UAGCACCAUUUGAAAUCGGUUA for Mm_miR-29c. Hs_RNU6-2_1 (Qiagen) was used as an internal control.

### In vitro experiments and transfection

Human HK-2 cells were used at passages 4 to 8 and cultured in epithelial basal media with growth factors and 10% serum. Human Angptl4- and Angptl3-specific siRNA (Invitrogen) were used at a concentration of 40 nM for 48 hours to effectively knock down Angptl4 and Angplt3. Cells were treated with or without TGFβ2 (10 ng/ml) for 48 hours and harvested for Western blot analysis. In a second set of experiments, isolated tubules from diabetic control and tmut mice were cocultured with podocytes and endothelial cells. Similarly, isolated podocytes from diabetic control and pmut mice were cocultured with tubules and endothelial cells. In another experiment, Human HK-2 cells were cultured in DMEM and Keratinocyte-SFM (1×) (Life Technologies Green Island NY) media, respectively. When the cells reached 70% confluence, recombinant human TGFβ1 (10 ng/ml) was placed in the serum-diluted medium for 48 hours.

For the transfection studies, HK-2 cells, which were maintained in serum-diluted media, were passaged in six-well plates. HK-2 cells were then transfected with 100 nM antagomir for miR-29a, miR-29b, and miR-29c (Qiagen), using Lipofectamine 2000 transfection reagent (Invitrogen, Carlsbad, CA, USA). The cells were incubated for 6 hours with Lipofectamine and the anti-microRNA complex in antibiotic-free media, after which the medium was refreshed before the cells were incubated for another 48 hours. Upon termination of incubation, total RNA was isolated using the miRNeasy Kit (Qiagen) following the manufacturer’s instructions for expression analysis, lipid uptake, and lipid oxidation.

### Luciferase assay

A luciferase assay was used to analyze the activity of the 3′UTR in human Angptl4. Amplified vector DNA Cloned 3′UTR human Angptl4 (Endofectin GeneCopoeia catalog no. EF013-S) and miRNA 3′UTR (MmiT088761-MT06, 264 ng/μl) were transfected into HK-2 cells. Transcriptional activity was evaluated by the Dual-Luciferase Reporter Assay System (Promega) in triplicate samples in the presence of control microRNA, mimetic, antagomiR, or inhibitor of 29s (100 nM).

### Fatty acid uptake

Cultured isolated kidney endothelial cells were incubated with medium containing 2 μCi [^14^C]-palmitate. [^14^C]-palmitate uptake was measured by liquid scintillation counting.

### Fatty acid oxidation

Cultured isolated kidney endothelial cells were incubated with medium containing 0.75 mM palmitate (conjugated to 2% free fatty acid–free BSA/[^14^C] palmitate at 2 μCi/ml) for 2 hours. The culture medium (1 ml) was transferred to a sealable tube, the cap of which housed a Whatman filter paper disc. ^14^CO_2_ trapped in the media was then released by acidification of media using 60% perchloric acid. Radioactivity that had become adsorbed onto the filter discs was quantified by liquid scintillation counting.

### Statistical analysis

All values are expressed as mean ± SEM and analyzed using GraphPad Prism 7 (GraphPad Software Inc., La Jolla, CA). One-way analysis of variance (ANOVA) followed by Tukey’s test and two-way ANOVA were used to analyze significance when comparing multiple independent groups. The post hoc tests were run only if F achieved *P* < 0.05 and there was no significant variance in homogeneity. In each experiment, *N* represents the number of separate experiments (in vitro) or the number of mice (in vivo). Technical replicates were used to ensure the reliability of single values. Data analyses were blinded. The data were considered statistically significant at *P* < 0.05.
