# Supplementary material for: Renal Angptl4 is a key fibrogenic molecule in progressive diabetic kidney disease
Source: Sci Adv. 2024 Dec 4;10(49):eadn6068. doi: 10.1126/sciadv.adn6068 (PMC11616692; doi:10.1126/sciadv.adn6068)
Supplement: Supplementary file 1 — Figs. S1 to S20 Tables S1 and S2 [file sciadv.adn6068_sm.pdf]

Science Advances
AAAS

Supplementary Materials for

Renal Angptl4 is a key fibrogenic molecule in progressive diabetic kidney disease

Swayam Prakash Srivastava *et al.*

Corresponding author: Swayam Prakash Srivastava, swayam.cdri@gmail.com;
Julie E. Goodwin, julie.goodwin@yale.edu

Sci. Adv. **10**, eadn6068 (2024)
DOI: 10.1126/sciadv.adn6068

This PDF file includes:

Figs. S1 to S20
Tables S1 and S2

Supplementary figure legends

Figure S1
mRNA array analysis in control and diabetic mice. Gene expression networking was analyzed using STRING (https://string-db.org/).

Figure S2
KEGG Pathway analysis, Gene Ontology (GO)-molecular function and GO-biological process analysis from the mRNA array data set.

Figure S3
A. Sirius red and PAS staining in kidneys of poloxamer-treated and vehicle-treated diabetic mice. Representative histologic images are shown (original magnification 300X). Relative area of fibrosis (%) was measured using ImageJ. Scale bar 50 μm. n=5/group. **B.** Plasma triglyceride measurement by calorimetry. n=5/group. **C.** Lipoprotein lipase (LPL) measurement. n=5/group. Data are mean ± SEM. Student t-test (unpaired two-tailed) was used for analysis of statistical significance. Significance- *$p<0.05$.

Figure S4
A. Relative gene expression analysis of Angplt3 and Angptl4 by qPCR in low-glucose (5.55 mM)- and high-glucose- (30 mM) treated cultured HK-2 cells. Data are mean ± SEM. Student t-test (unpaired two-tailed) was used for analysis of statistical significance. **B.** Relative gene expression of *FSP-1*, *Vimentin* and *Collagen I* by qPCR in the scrambled control, scrambled transfected TGFβ1-stimulated (10 ng/ml) and TGFβ1-stimulated with (10 ng/ml) Angplt4 or Angptl3 siRNA-transfected cultured HK-2 cells. Data are mean ± SEM. One-way Anova with Tukey's multiple comparison post hoc test was used to calculate statistical significance. **C.** Analysis of human ANGPTL4 expression in different tissues using Genotype-Tissue Expression (GTEx) database. TPM (transcript per million reads) is shown.

Figure S5
A. Immunofluorescence analysis of β-gal/DAPI in kidneys of control and Angptl4$^{-/-}$ mice. n=5/group. β-gal FITC-labeled and DAPI (nuclei, blue). Representative images (magnification 300X) are shown; three independent experiments were analyzed. Scale bar 50 μm. Gross histology showing Lac-Z staining in Angptl4$^{-/-}$ kidneys and X-gal staining by light microscopy in Angptl4$^{-/-}$ kidney sections. **B.** Masson Trichrome (MTS), Sirius red and PAS staining in kidneys of nondiabetic control and nondiabetic Angptl4$^{-/-}$ mice shown at low magnification. Representative histologic images are shown (original magnification 200X). Relative area of fibrosis (%), relative collagen deposition (%) and glomerular surface area were measured using ImageJ. Scale bar 100 μm. n=5/group. **C.** Masson Trichrome (MTS), Sirius red and PAS staining in kidneys from diabetic control and diabetic Angptl4$^{-/-}$ mice (original magnification 50X). n=5/group. **D.** Relative gene expression of *αSMA* and *Collagen I* by qRT PCR. n=6-7/per group. 18S was used as internal control. Data are mean ± SEM. Student t-test (unpaired two-tailed) was used for analysis of statistical significance.

Figure S6

A. In the UUO model, left kidneys were ligated in control littermates and Angptl4 mutant mice. Two days after UUO, four consecutive, low-dose (50 mg/kg ip) STZ injections were given. On day 11 kidneys were excised. Masson trichrome staining (MTS) in the contralateral non-operated kidney and UUO-operated kidneys in control and Angptl4 mutant mice were analyzed. Representative histologic images are shown; (original magnification 300X). Area of fibrosis (%) was measured using ImageJ program. n=6 mice/group combined from two independent experiments. Scale bar 100 μm. **B.** Blood glucose and kidney weight/body weight measurement in the indicated groups. Data are mean ± SEM. One-way Anova with Tukey's multiple comparison post hoc test was used to calculate statistical significance. Significance-*p<0.05. **C.** Basal oxygen consumption rates (OCR) in kidneys from non-diabetic and diabetic wild-type and Angptl4$^{-/-}$ mice were measured using the Seahorse Flux Analyzer (Agilent) according to the manufacturer's instructions. Respiration rates were measured three times using an instrument protocol of 3-minute mix, 2-minute wait, and 3-minute measure. Flux rates were normalized to tissue weight. Experiments were repeated in 3 mice/condition. Data are mean ± SEM. One-way Anova with Tukey's multiple comparison post hoc test was used to calculate statistical significance. Significance-*p<0.05.

Figure S7

A. Oil Red O staining in kidneys from control and diabetic Angptl4$^{-/-}$ mice. N=5/group. Scale bar 50 μm. Representative pictures are shown; original magnification 300X **B.** Kidney triglycerides. N=5/group **C.** Kidney ATP levels N=5/group. Data are mean ± SEM. One-way Anova with Tukey's multiple comparison post hoc test was used for analysis of statistical significance. Significance-*p<0.05. **D.** *FASN* mRNA expression was analyzed by real-time PCR in control and diabetic Angptl4$^{-/-}$ mice. N=6-7/group. 18S was used as an internal control Data are mean ± SEM. Student t-test (unpaired two-tailed) was used for analysis of statistical significance. Significance-*p<0.05.

Figure S8

A. Immunofluorescence analysis of β-gal/DAPI in kidneys from control and pmut mice. n=5/group. β-gal FITC-labeled and DAPI (nuclei, blue). Representative images are shown. **B.** Relative gene expression analysis of *Angptl4* in isolated podocytes from control and pmut mice. Data are mean ± SEM. n=7/group. Student's t-test (unpaired two-tailed) was used for analysis of statistical significance. Significance *p<0.05. **C.** Sirius red and PAS staining in kidneys from nondiabetic and diabetic, control and pmut mice at low magnification (original magnification 50X). n=7/group. **D.** *αSMA* and *Collagen I* mRNA expression in kidneys from nondiabetic and diabetic, control and pmut mice. N=6/group. 18S was used as an internal control. Data are mean ± SEM. One-way Anova with Tukey's multiple comparison post hoc test was used for analysis of statistical significance. Significance-*p<0.05.

Figure S9

A. Oil Red O staining in kidneys from control and diabetic pmut mice. N=5/group. Representative pictures are shown. Scale bar 50 μm. Original magnification 300X **B.** Kidney triglycerides. N=5/group. **C.** Lipid uptake was analyzed and is shown as fold change. CPM was measured and was normalized by protein. N=5/group **D.**

[14]CPalmitate oxidation was measured and was normalized by protein. N=5/group **E.** Kidney ATP levels N=5/group. **F.** *FASN* mRNA expression was analyzed in control and diabetic pmut. N=6/group. 18S was used as an internal control **F.** Kidney total cholesterol levels. N=5/group. **G.** Graphical representation of co-culture experiments: endothelial cells were co-cultured with podocytes isolated from diabetic control or diabetic pmut mice. **H.** qPCR gene expression analysis of *αSMA*, *FSP-1* and *TGFβR1* in endothelial cells that were co-cultured with podocytes from control mice or pmut mice**. I.** Measurement of IL-1β, IL-6, IL-10, IL-17 and TNFα levels in the cell supernatant. **J.** Radiolabeled [^{14}C]palmitate uptake and oxidation, were measured in indicated groups. CPM of each sample was counted. **K.** ATP measurement in the endothelial cells which were co-cultured with podocytes either from diabetic control or diabetic pmut mice. **L.** Endothelial cell permeability assay was analyzed by measuring FITC-dextran**.** Data are mean ± SEM. Student t-test (unpaired two-tailed) was used for analysis of statistical significance. Significance- *p<0.05.

Figure S10
A. Schematic diagram showing high fat diet (HFD) treatment in control and pmut mice. HFD was provided for 16 weeks to control and pmut mice. **B-E.** Physiological parameters such as body weight, blood glucose, albumin-to-creatinine ratio (ACR) and kidney weight were measured. N=5/group. **F.** Representative histologic images from MTS, Sirius red and PAS staining in kidneys of HFD-fed control and pmut mice are shown. Original magnification 300X. Relative area of fibrosis (%), relative collagen deposition (%) and glomerular surface area were measured using ImageJ. N=5/group. Scale bar 50 μm. Data are mean ± SEM. One-way Anova with Tukey's multiple comparison post hoc test was used to calculate statistical significance. Significance- *p<0.05.

Figure S11
A. Immunofluorescence analysis of β-gal/DAPI in kidneys of control and tmut mice. n=5/group. β-gal FITC-labeled and DAPI (nuclei, blue). Representative images are shown. **B.** Relative gene expression analysis of *Angptl4* in isolated tubules from control and tmut mice. Data are mean ± SEM. n=6/group. Student t-test (unpaired two-tailed) was used for analysis of statistical significance. Significance *p<0.05. **C**. Sirius red and PAS staining in kidneys of nondiabetic and diabetic control and tmut mice at low magnification (original magnification 50X). n=6/group. **D.** *αSMA and Collagen I* mRNA expression in kidneys of nondiabetic and diabetic, control and tmut mice. N=6/group. 18S was used as internal control to normalize the data. Data are mean ± SEM. One-way Anova with Tukey's multiple comparison post hoc test was used for analysis of statistical significance. Significance-*p<0.05.

Figure S12
A. Oil Red O staining in kidneys from control and diabetic tmut mice. N=5/group. Representative pictures are shown. Scale bar 50 μm. Original magnification 300X **B.** Kidney triglycerides. N=5/group. **C.** Lipid uptake was analyzed and is shown as fold change. CPM was measured and was normalized by protein. N=5/group **D.** [^{14}C]Palmitate oxidation was measured and was normalized to protein. N=5/group **E.** Kidney ATP levels N=5/group. **F.** *FASN* mRNA expression levels were analyzed by real-time PCR in the control and diabetic tmut mice. N=6/group. 18S was used as an

internal control. **G.** Kidney total cholesterol levels. n=5/group. **H.** Graphical representation of co-culture experiments in endothelial cells which were co-cultured with tubules isolated from diabetic control or diabetic tmut mice. **I**. qPCR gene expression analysis of *αSMA*, *FSP-1* and *TGFβR1* in endothelial cells which were co-cultured with tubules from control mice and tmut mice. **J**. Measurement of IL-1β, IL-6, IL-10, IL-17 and TNFα levels in the cell supernatant. **K.** Radiolabeled [^{14}C]palmitate uptake and oxidation, were measured in indicated groups. CPM of each sample was counted. **L**. ATP measurement in endothelial cells which were co-cultured with tubules either from diabetic control or diabetic tmut mice. **M.** Endothelial permeability assay was performed by measuring the concentration of FITC-dextran. Data are mean ± SEM. Student t-test (unpaired two-tailed) was used for analysis of statistical significance. Significance- *p<0.05.

Figure S13
A. Schematic diagram showing high fat diet (HFD) treatment in control and tmut mice. HFD was fed for 16 weeks. **B-E.** Physiological parameters such as body weight, blood glucose, albumin-to-creatine ratio (ACR) and kidney weight were measured. N=5/group. **F.** Representative histologic images from MTS, Sirius red and PAS staining in kidneys of HFD-fed control and tmut mice are shown. Original magnification 300X. Relative area of fibrosis (%), relative collagen deposition (%) and glomerular surface area were measured using ImageJ. N=5/group. Scale bar 50 *μ*m. Data are mean ± SEM. One-way Anova with Tukey's multiple comparison post hoc test was used to calculate statistical significance. Significance-*p<0.05.

Figure S14
A. Duo-link proximity ligation assay of DPP-4/β1-Integrin in podocytes and tubules of non-diabetic and diabetic, pmut and tmut mice. n=5/group. **B.** Duo-link proximity ligation assay of Angptl4/β1-Integrin in siRNA-β1-Integrin and siRNA-DPP-4-tranfected podocytes and HK-2 cells treated with or without TGFβ1 (10 ng/ml). Experiments were performed in triplicate **C.** Duo-link proximity ligation assay of Angptl4/DPP-4 in siRNA-β1-Integrin and siRNA-DPP-4-tranfected podocytes and HK-2 cells treated with or without TGFβ1 (10 ng/ml). Experiments were performed in triplicate

Figure S15
Immunohistochemical analysis of, DPP-4, TGFβ1 and p-smad3 in kidneys from diabetic controls and diabetic pmut and tmut mice. Scale bar 50 μm. n=5 mice/group. Representative micrographic images (original magnification 400X) are shown.

Figure S16
A. *Angptl4* mRNA expression in kidneys from linagliptin-treated diabetic mice and untreated diabetic mice. n=7/group. **B.** *Angptl4* mRNA expression in kidneys of neutralizing integrin β1 antibody (N-integrin β1)-injected diabetic mice and IgG-injected diabetic mice. n=5/group. Data are mean ± SEM. Student t-test (unpaired two-tailed) was used for analysis of statistical significance. Significance-*p<0.05. **C.** Physiological characteristics including blood glucose, kidney weight/body weight and albumin-to-creatine ratio were analyzed in control LNA- and miR-29 LNA-treated nondiabetic and diabetic mice. n=7 in the nondiabetic group and n=6 in the diabetic

group. **D.** Relative gene expression analysis of miR-29a-3p, miR-29b-3p, and miR-29c-3p in the kidneys of control LNA- and miR-29 LNA-treated nondiabetic and diabetic mice. Data are mean ± SEM. n=7 in the nondiabetic group and n=6 in the diabetic group. One-way Anova with Tukey's multiple comparison post hoc test was used to calculate statistical significance. Significance-*p<0.05.

Figure S17
A. Physiological characteristics including body weight, blood glucose, kidney weight/body weight, albumin-to-creatine ratio and mean blood pressure were analyzed in control, diabetic and N-acetyl-D-mannosamine-treated diabetic mice. n=6/group. **B.** Representative histologic images from MTS, Sirius red and PAS staining in kidneys of control, diabetic and N-acetyl-D-mannosamine-treated diabetic mice (original magnification 300X). Relative area of fibrosis (%), relative collagen deposition(%) and glomerular surface area were measured using ImageJ. Scale bar 70 *μ*m. Data are mean ± SEM. One-way Anova with Tukey's multiple comparison post hoc test was used to calculate statistical significance. Significance-*p<0.05.

Figure S18
A-B. Plasma levels of IL-1β, IL-5, IL-6, CCL3, CCL5, and Mo-GCSF from nondiabetic and diabetic pmut and tmut mice were analyzed by cytokine array analysis. n=5 mice/group. Data are mean ± SEM. One-way Anova with Tukey's multiple comparison test was used to calculate statistical significance. Significance- *p<0.05. **C-D.** Kidney IL-1β, IL-6 and MCP-1 levels in nondiabetic and diabetic pmut and tmut mice. N=5 mice/group. Data are mean ± SEM. One-way Anova with Tukey's multiple comparison test was used to calculate statistical significance. Significance- *p<0.05.

Figure S19
A. Immunofluorescence analysis of p-IKB/DAPI in isolated podocytes and tubules from nondiabetic pmut and tmut mice. n=5/group. p-IKB, rhodamine-labeled and DAPI (nuclei, blue). Representative images are shown **B.** Immunohistochemical analysis of p-65 in kidneys of nondiabetic pmut and tmut mice. Representative merged (original magnification 400X) images are shown. Scale bar 50 μm. n=5 mice/group. **C**. Immunofluorescence analysis of p-65/DAPI in isolated podocytes and tubules from nondiabetic pmut and tmut mice. n=5/group. p-65, FITC-labeled and DAPI (nuclei, blue). Representative images are shown; three independent experiments were analyzed.

Figure S20
Representative histologic images from Sirius red staining in kidneys from control, angiotensin-converting enzyme inhibitor imidapril (ACEi)-treated, N-seryl-acetyl-lysyl-proline (AcSDKP)-treated, combined ACEi+AcSDKP-treated 2-DG-treated, DCA-treated, fenofibrate-treated or simvastatin-treated diabetic mice. Scale bar 50 *μ*m. Quantification of collagen deposition and relative gene expression levels of Angptl4 are provided for each treatment. Data are mean ± SEM. One-way Anova with Tukey's multiple comparison post hoc test was used to calculate statistical significance. Significance-*p<0.05.

Table S1. Mouse primers

Table S2. Human primers

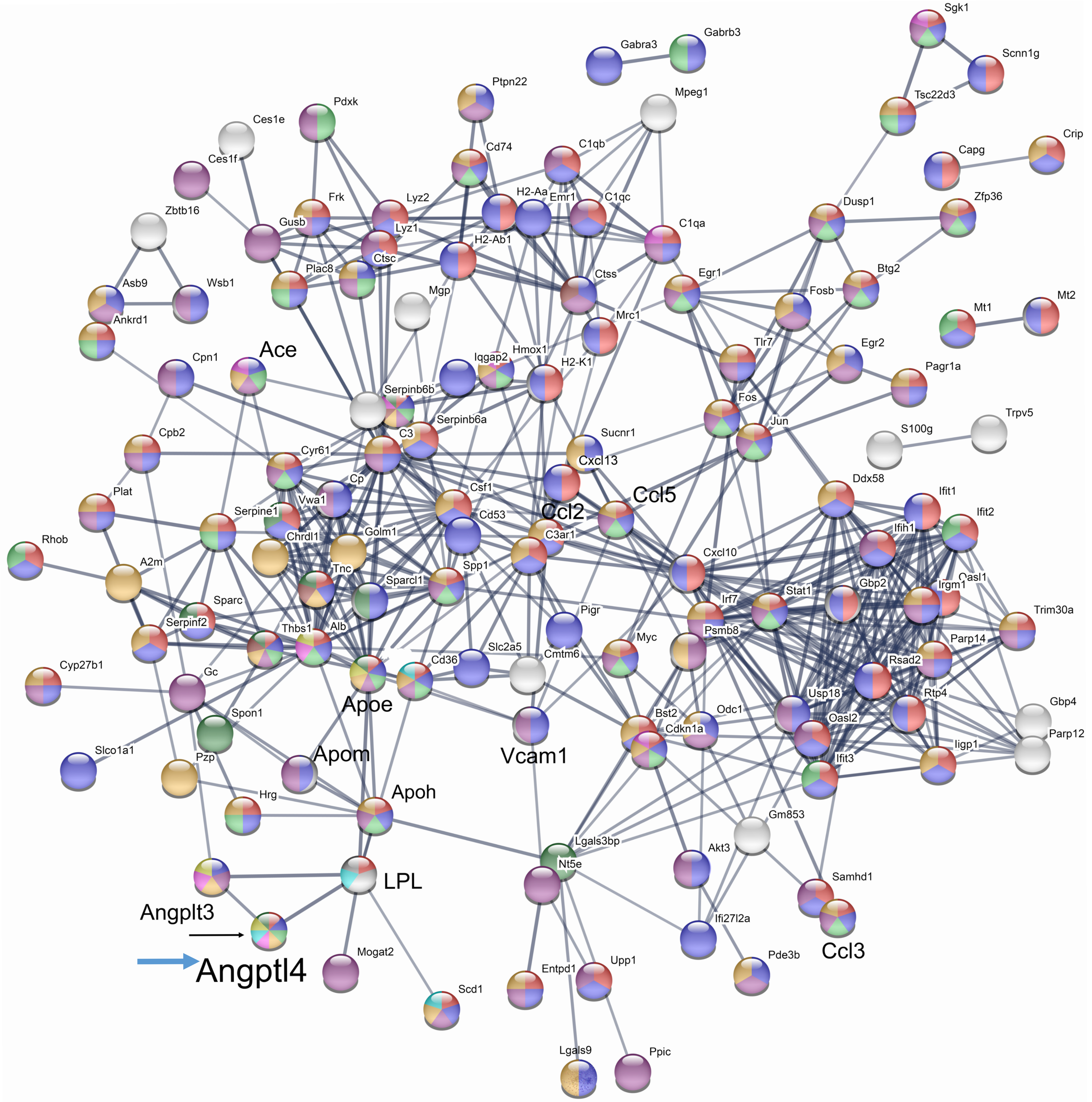
Sgk1
Gabra3
Gabrb3
Scnn1g
Tsc22d3
Ptpn22
Mpeg1
Pdxk
Ces1e
Crip
Capg
Cd74
C1qb
Ces1f
Zbtb16
Frk
Lyz2
H2-Aa
Emr1
C1qc
Dusp1
Zfp36
Gusb
Lyz1
C1qa
H2-Ab1
Plac8
Ctsc
Ctss
Btg2
Asb9
Wsb1
Mgp
Egr1
Fosb
Mt1
Mt2
Ankrd1
Mrc1
Ace
Tlr7
Egr2
Cpn1
Hmox1
Iqgap2
H2-K1
Pagr1a
Serpinb6b
Fos
Trpv5
Serpinb6a
S100g
Jun
C3
Sucnr1
Cpb2
Cyr61
Cxcl13
Cp
Ddx58
Plat
Csf1
Ccl5
Ifit1
Vwa1
Ifit2
Serpine1
Ccl2
Cd53
Ifih1
Chrdl1
Golm1
C3ar1
Rhob
Cxcl10
Oasl1
A2m
Tnc
Irgm1
Spp1
Sparcl1
Stat1
Gbp2
Sparc
Irf7
Trim30a
Pigr
Serpinf2
Thbs1
Alb
Myc
Psmb8
Parp14
Slc2a5
Cmtm6
Cyp27b1
Cd36
Rsad2
Gc
Rtp4
Usp18
Gbp4
Apoe
Odc1
Oasl2
Spon1
Bst2
Cdkn1a
Parp12
Slco1a1
Apom
Vcam1
Iigp1
Pzp
Ifit3
Apoh
Hrg
Gm853
Lgals3bp
Akt3
Nt5e
Samhd1
LPL
Angplt3
Ifi27l2a
Ccl3
Pde3b
Mogat2
Angptl4
Upp1
Entpd1
Scd1
Lgals9
Ppic

Figure S1

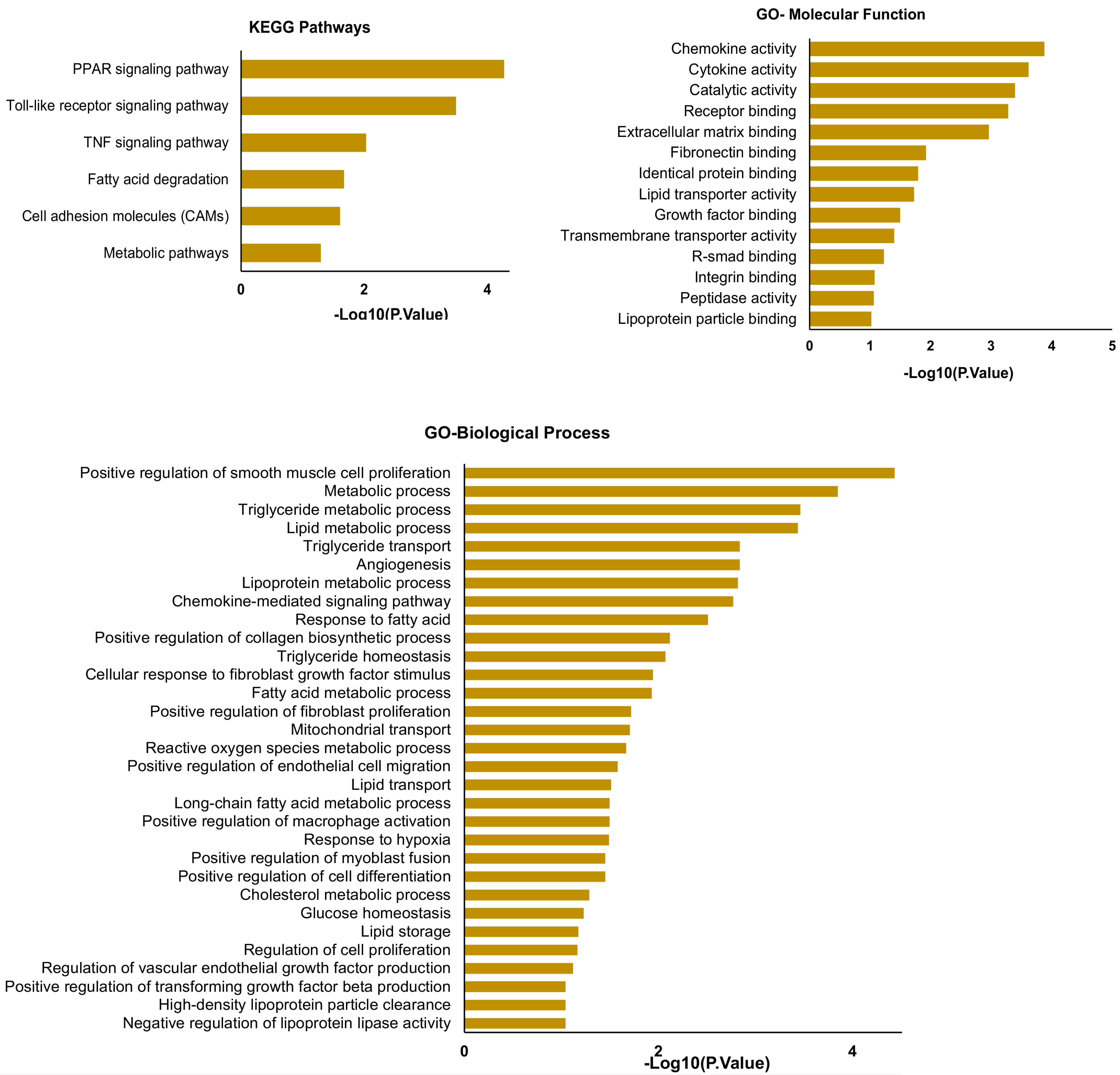
KEGG Pathways
PPAR signaling pathway
Toll-like receptor signaling pathway
TNF signaling pathway
Fatty acid degradation
Cell adhesion molecules (CAMs)
Metabolic pathways
0
2
4
-Log10(P.Value)
GO- Molecular Function
Chemokine activity
Cytokine activity
Catalytic activity
Receptor binding
Extracellular matrix binding
Fibronectin binding
Identical protein binding
Lipid transporter activity
Growth factor binding
Transmembrane transporter activity
R-smad binding
Integrin binding
Peptidase activity
Lipoprotein particle binding
0
1
2
3
4
5
-Log10(P.Value)
GO-Biological Process
Positive regulation of smooth muscle cell proliferation
Metabolic process
Triglyceride metabolic process
Lipid metabolic process
Triglyceride transport
Angiogenesis
Lipoprotein metabolic process
Chemokine-mediated signaling pathway
Response to fatty acid
Positive regulation of collagen biosynthetic process
Triglyceride homeostasis
Cellular response to fibroblast growth factor stimulus
Fatty acid metabolic process
Positive regulation of fibroblast proliferation
Mitochondrial transport
Reactive oxygen species metabolic process
Positive regulation of endothelial cell migration
Lipid transport
Long-chain fatty acid metabolic process
Positive regulation of macrophage activation
Response to hypoxia
Positive regulation of myoblast fusion
Positive regulation of cell differentiation
Cholesterol metabolic process
Glucose homeostasis
Lipid storage
Regulation of cell proliferation
Regulation of vascular endothelial growth factor production
Positive regulation of transforming growth factor beta production
High-density lipoprotein particle clearance
Negative regulation of lipoprotein lipase activity
0
2
4
-Log10(P.Value)

Figure S2

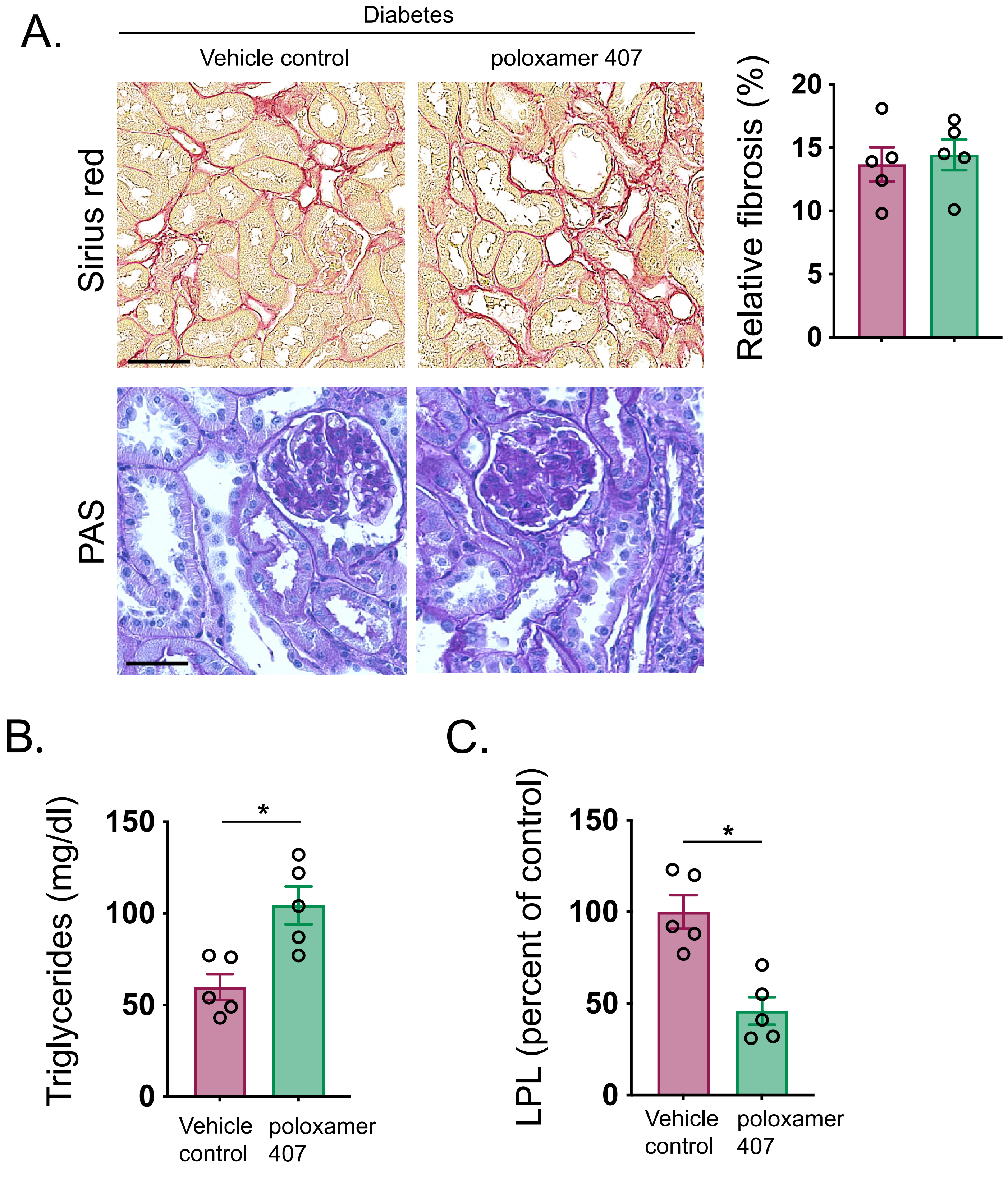
A.
Diabetes
Vehicle control
poloxamer 407
Sirius red
PAS
Relative fibrosis (%)
20
15
10
5
0
B.
Triglycerides (mg/dl)
150
100
50
0
*
Vehicle control
poloxamer 407
C.
LPL (percent of control)
150
100
50
0
*
Vehicle control
poloxamer 407

Figure S3

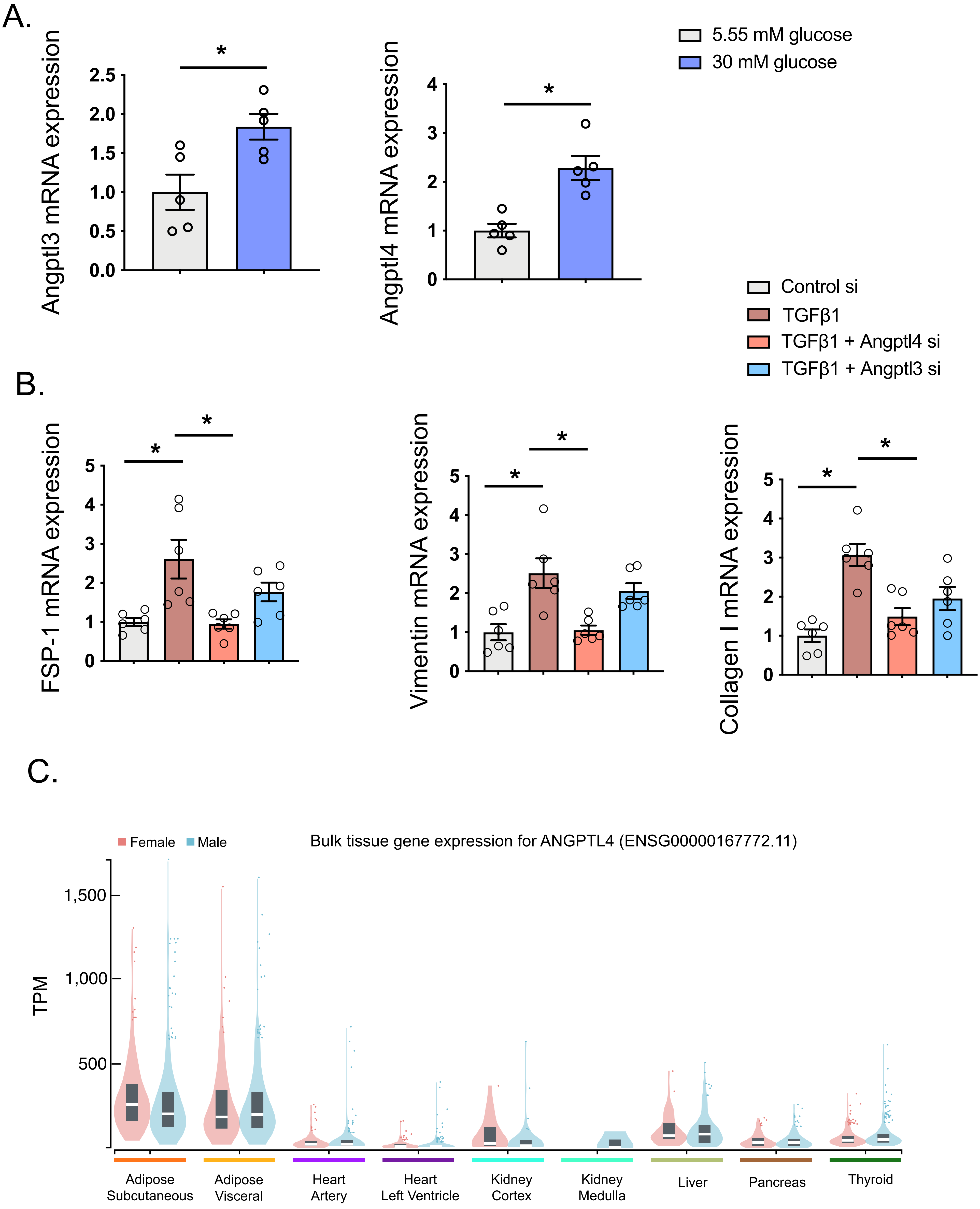
A.
5.55 mM glucose
30 mM glucose
Angptl3 mRNA expression
Angptl4 mRNA expression
*
B.
Control si
TGFβ1
TGFβ1 + Angptl4 si
TGFβ1 + Angptl3 si
FSP-1 mRNA expression
Vimentin mRNA expression
Collagen I mRNA expression
C.
Female
Male
Bulk tissue gene expression for ANGPTL4 (ENSG00000167772.11)
TPM
1,500
1,000
500
Adipose Subcutaneous
Adipose Visceral
Heart Artery
Heart Left Ventricle
Kidney Cortex
Kidney Medulla
Liver
Pancreas
Thyroid

Figure S4

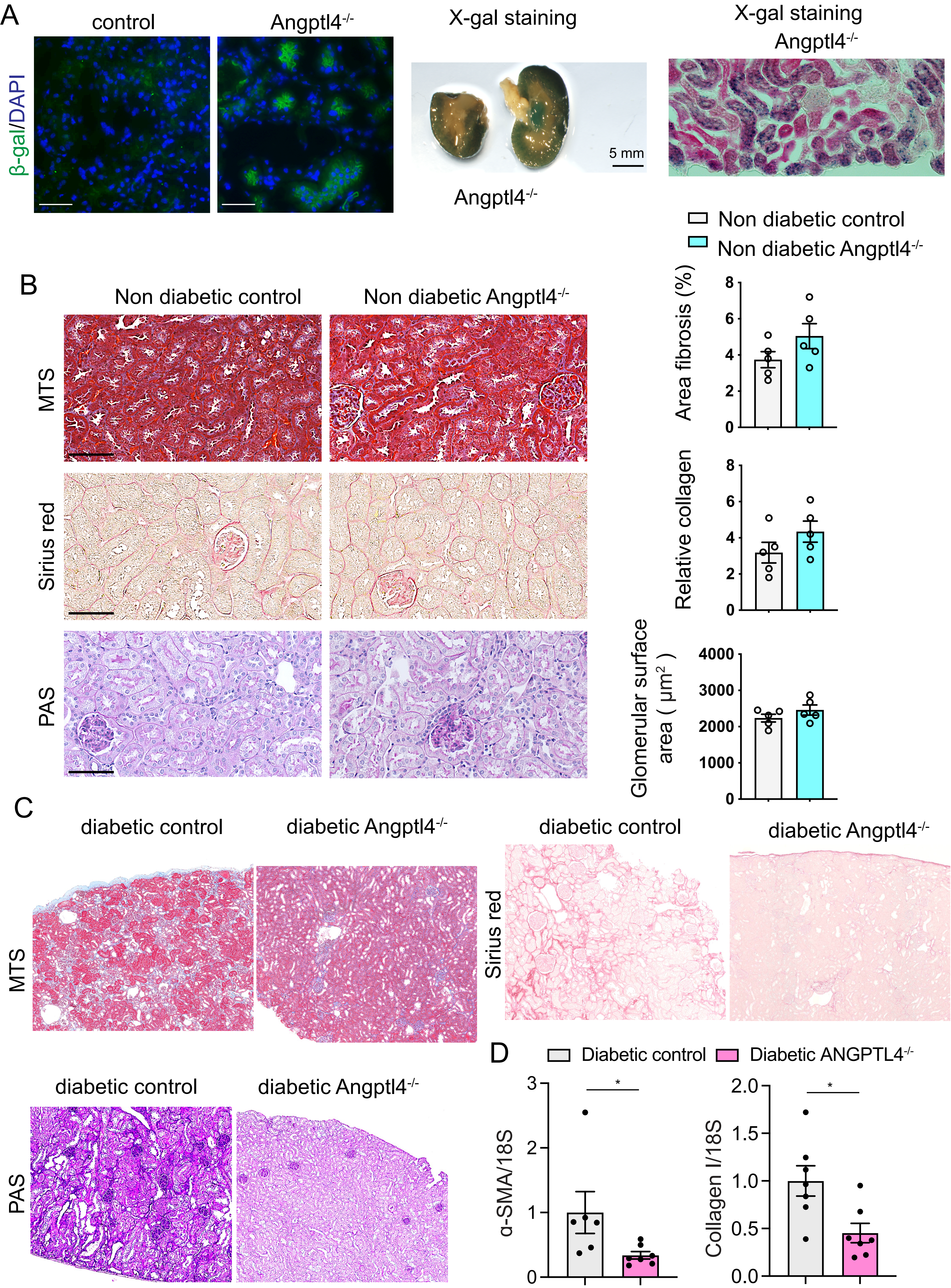

A
control
Angptl4-/-
β-gal/DAPI
X-gal staining
5 mm
Angptl4-/-
X-gal staining
Angptl4-/-
Non diabetic control
Non diabetic Angptl4-/-
B
Non diabetic control
Non diabetic Angptl4-/-
MTS
Sirius red
PAS
Area fibrosis (%)
Relative collagen
Glomerular surface area (μm²)
C
diabetic control
diabetic Angptl4-/-
MTS
Sirius red
PAS
D
Diabetic control
Diabetic ANGPTL4-/-
α-SMA/18S
Collagen I/18S
*

Figure S5

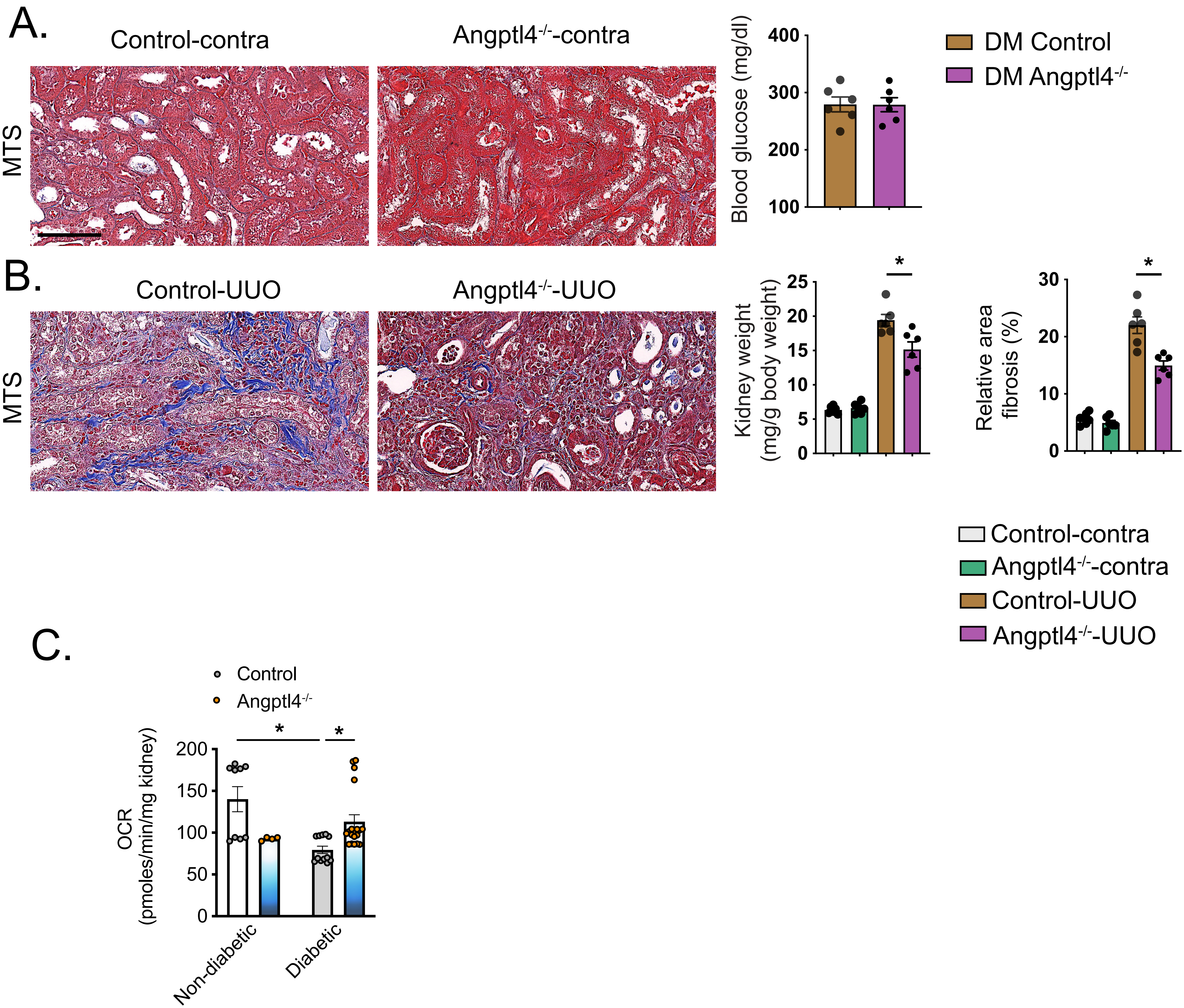
A.
Control-contra
Angptl4-/--contra
MTS
Blood glucose (mg/dl)
400
300
200
100
DM Control
DM Angptl4-/-
B.
Control-UUO
Angptl4-/--UUO
MTS
Kidney weight (mg/g body weight)
25
20
15
10
5
0
*
Relative area fibrosis (%)
30
20
10
0
*
Control-contra
Angptl4-/--contra
Control-UUO
Angptl4-/--UUO
C.
Control
Angptl4-/-
OCR (pmoles/min/mg kidney)
200
150
100
50
0
*
*
Non-diabetic
Diabetic

Figure S6

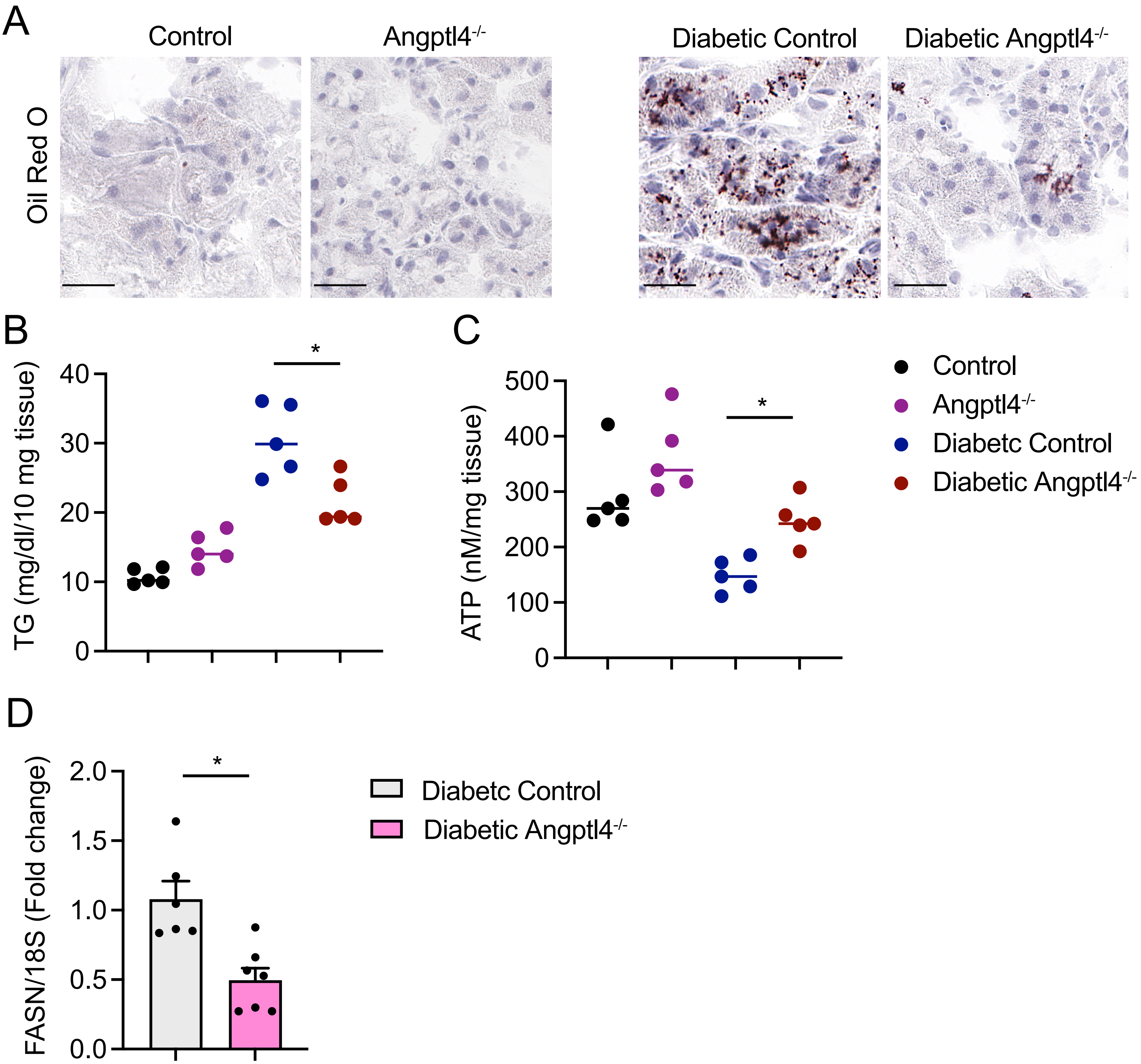

A
Control
Angptl4-/-
Diabetic Control
Diabetic Angptl4-/-
Oil Red O
B
TG (mg/dl/10 mg tissue)
40
30
20
10
0
*
C
ATP (nM/mg tissue)
500
400
300
200
100
0
*
Control
Angptl4-/-
Diabetc Control
Diabetic Angptl4-/-
D
FASN/18S (Fold change)
2.0
1.5
1.0
0.5
0.0
*
Diabetc Control
Diabetic Angptl4-/-

Figure S7

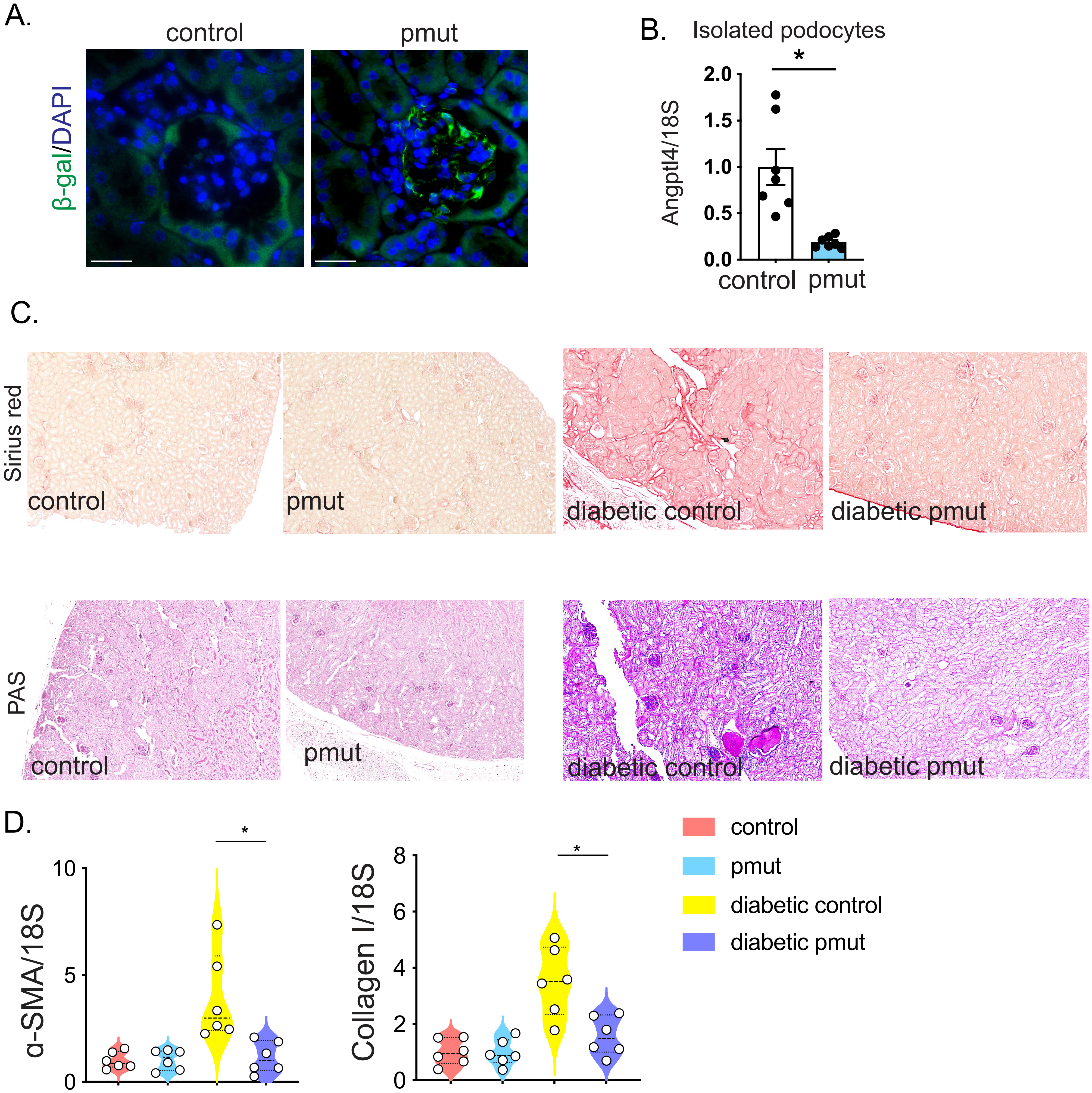
A.
control
pmut
β-gal/DAPI
B.
Isolated podocytes
*
Angptl4/18S
2.0
1.5
1.0
0.5
0.0
control
pmut
C.
Sirius red
control
pmut
diabetic control
diabetic pmut
PAS
control
pmut
diabetic control
diabetic pmut
D.
α-SMA/18S
10
5
0
*
Collagen I/18S
8
6
4
2
0
*
control
pmut
diabetic control
diabetic pmut

Figure S8

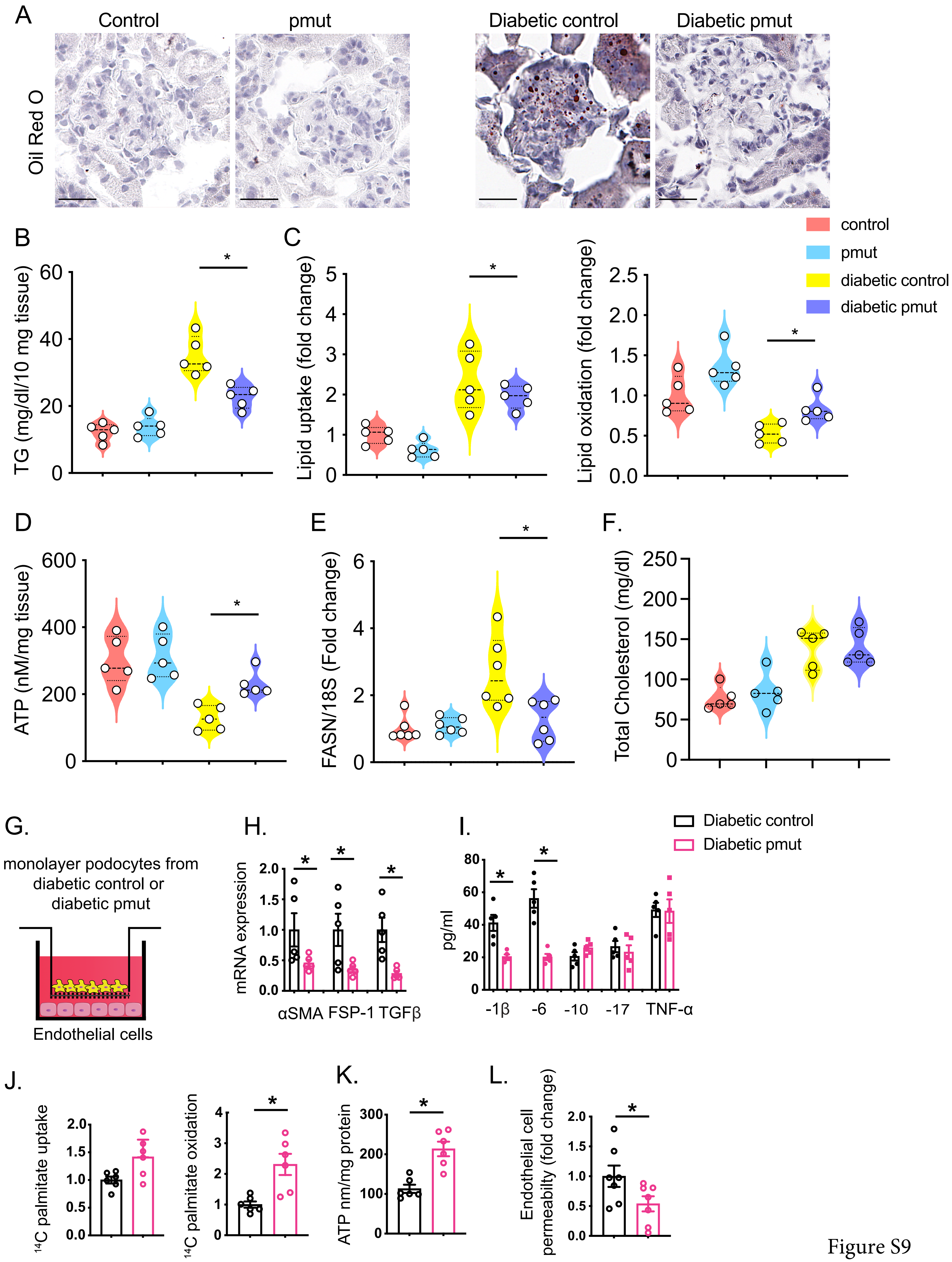
A
Control
pmut
Diabetic control
Diabetic pmut
Oil Red O
B
TG (mg/dl/10 mg tissue)
C
Lipid uptake (fold change)
Lipid oxidation (fold change)
control
pmut
diabetic control
diabetic pmut
D
ATP (nM/mg tissue)
E
FASN/18S (Fold change)
F.
Total Cholesterol (mg/dl)
G.
monolayer podocytes from diabetic control or diabetic pmut
Endothelial cells
H.
mRNA expression
αSMA FSP-1 TGFβ
I.
pg/ml
-1β
-6
-10
-17
TNF-α
Diabetic control
Diabetic pmut
J.
14C palmitate uptake
14C palmitate oxidation
K.
ATP nm/mg protein
L.
Endothelial cell permeability (fold change)

Figure S9

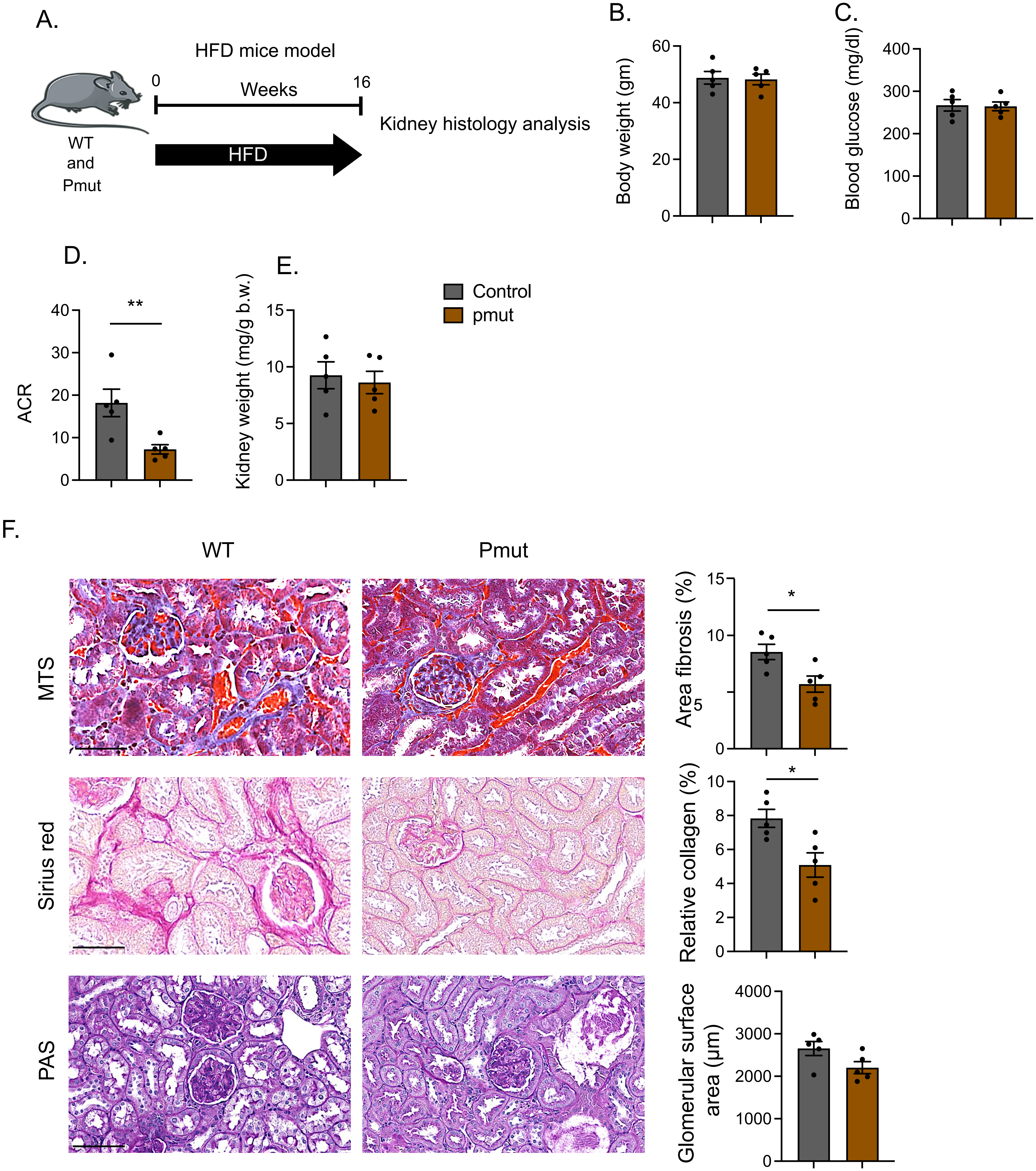
A.
HFD mice model
0
Weeks
16
WT
and
Pmut
HFD
Kidney histology analysis
B.
Body weight (gm)
60
40
20
0
C.
Blood glucose (mg/dl)
400
300
200
100
0
D.
ACR
**
40
30
20
10
0
E.
Kidney weight (mg/g b.w.)
15
10
5
0
Control
pmut
F.
WT
Pmut
MTS
Sirius red
PAS
Area fibrosis (%)
*
15
10
5
0
Relative collagen (%)
*
10
8
6
4
2
0
Glomerular surface area (μm)
4000
3000
2000
1000
0

Figure S10

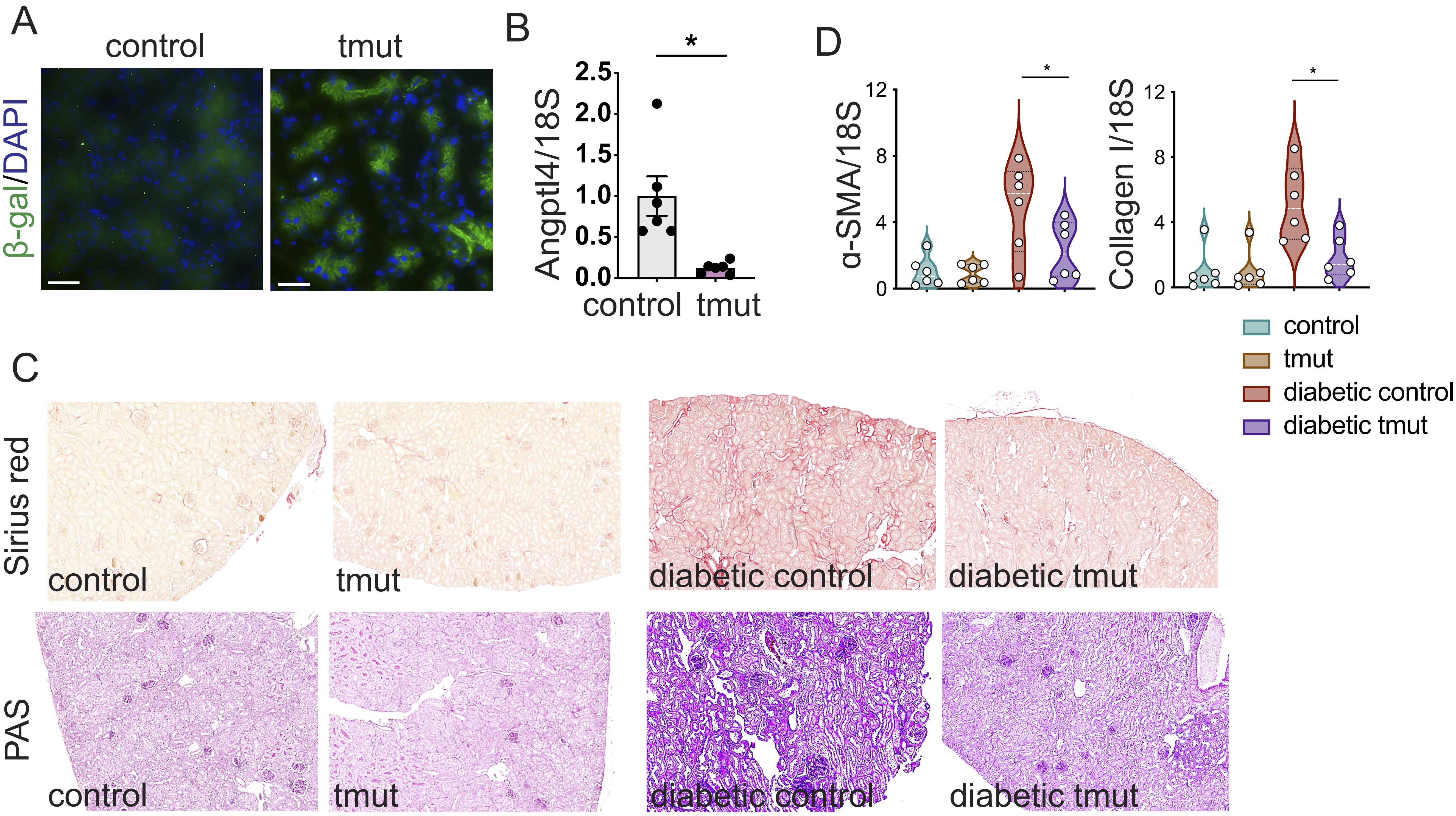
A
control
tmut
β-gal/DAPI
B
Angptl4/18S
2.5
2.0
1.5
1.0
0.5
0.0
*
control
tmut
D
α-SMA/18S
12
8
4
0
*
Collagen I/18S
12
8
4
0
*
control
tmut
diabetic control
diabetic tmut
C
Sirius red
control
tmut
diabetic control
diabetic tmut
PAS
control
tmut
diabetic control
diabetic tmut

Figure S11

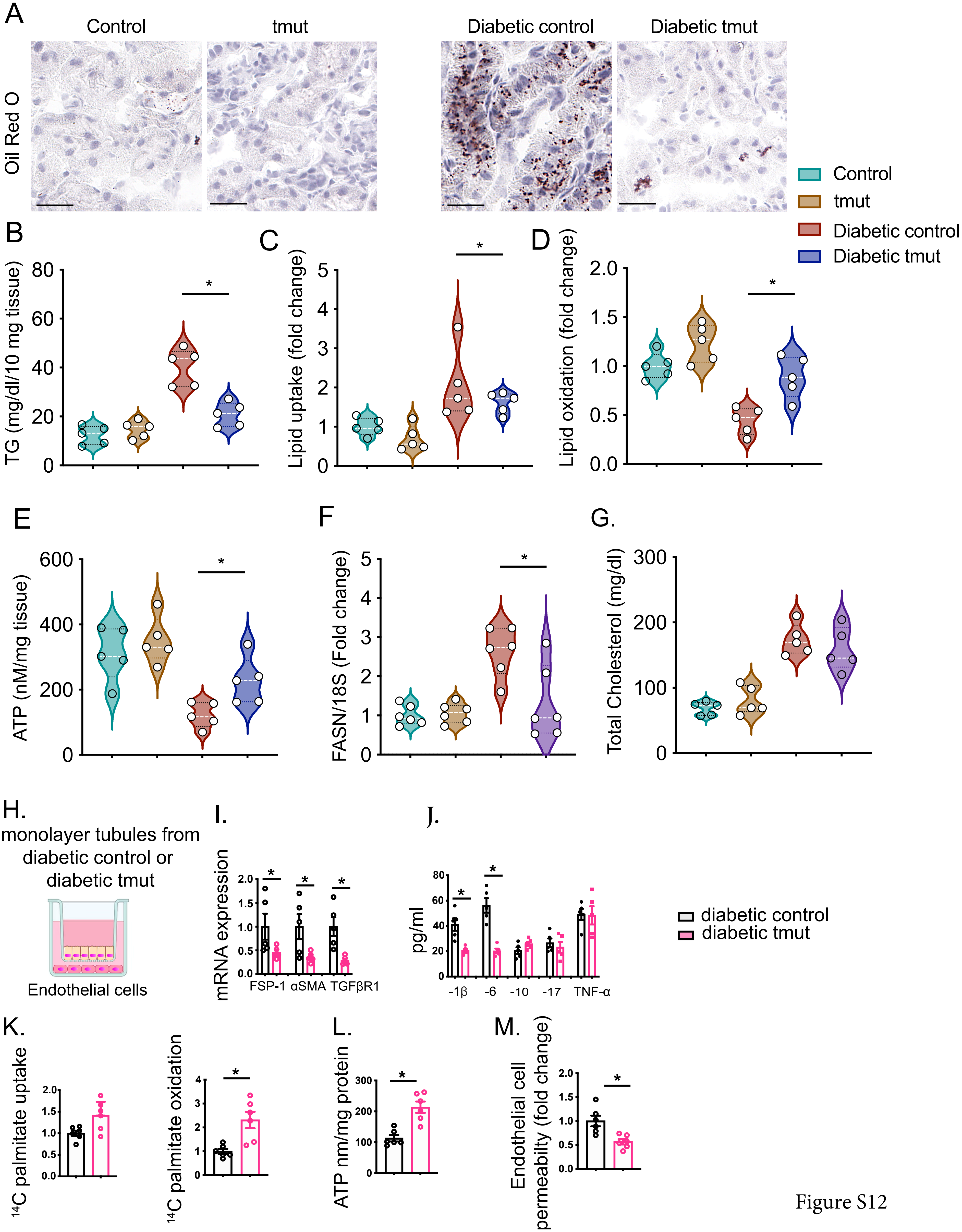
A
Control
tmut
Diabetic control
Diabetic tmut
Oil Red O
Control
tmut
Diabetic control
Diabetic tmut
B
TG (mg/dl/10 mg tissue)
C
Lipid uptake (fold change)
D
Lipid oxidation (fold change)
E
ATP (nM/mg tissue)
F
FASN/18S (Fold change)
G.
Total Cholesterol (mg/dl)
H.
monolayer tubules from diabetic control or diabetic tmut
Endothelial cells
I.
mRNA expression
FSP-1
αSMA
TGFβR1
J.
pg/ml
-1β
-6
-10
-17
TNF-α
diabetic control
diabetic tmut
K.
14C palmitate uptake
14C palmitate oxidation
L.
ATP nm/mg protein
M.
Endothelial cell permeabilty (fold change)

Figure S12

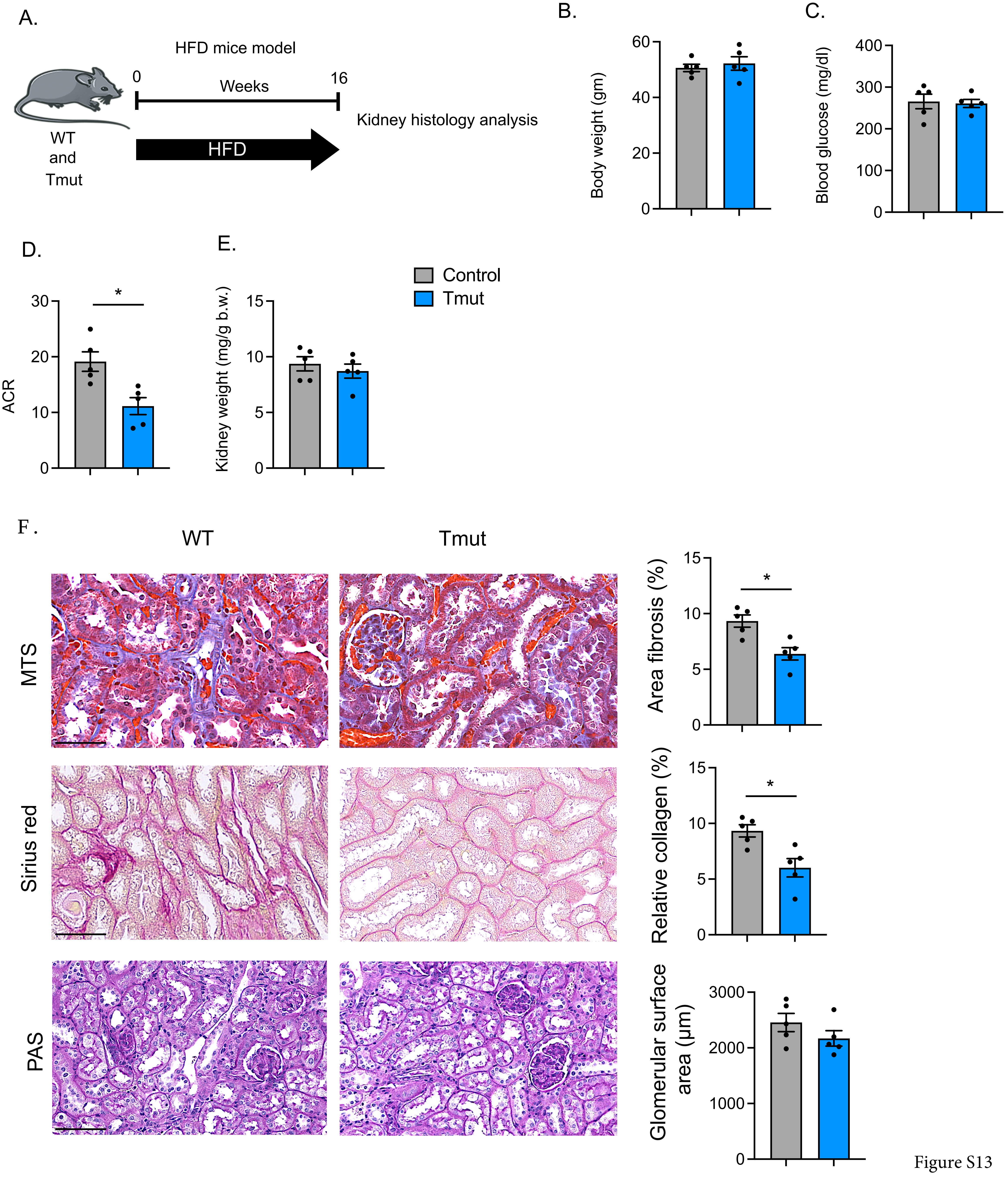
A.
HFD mice model
0
Weeks
16
WT
and
Tmut
HFD
Kidney histology analysis
B.
Body weight (gm)
C.
Blood glucose (mg/dl)
D.
ACR
E.
Kidney weight (mg/g b.w.)
Control
Tmut
F.
WT
Tmut
MTS
Sirius red
PAS
Area fibrosis (%)
Relative collagen (%)
Glomerular surface area (µm)

Figure S13

Figure S14

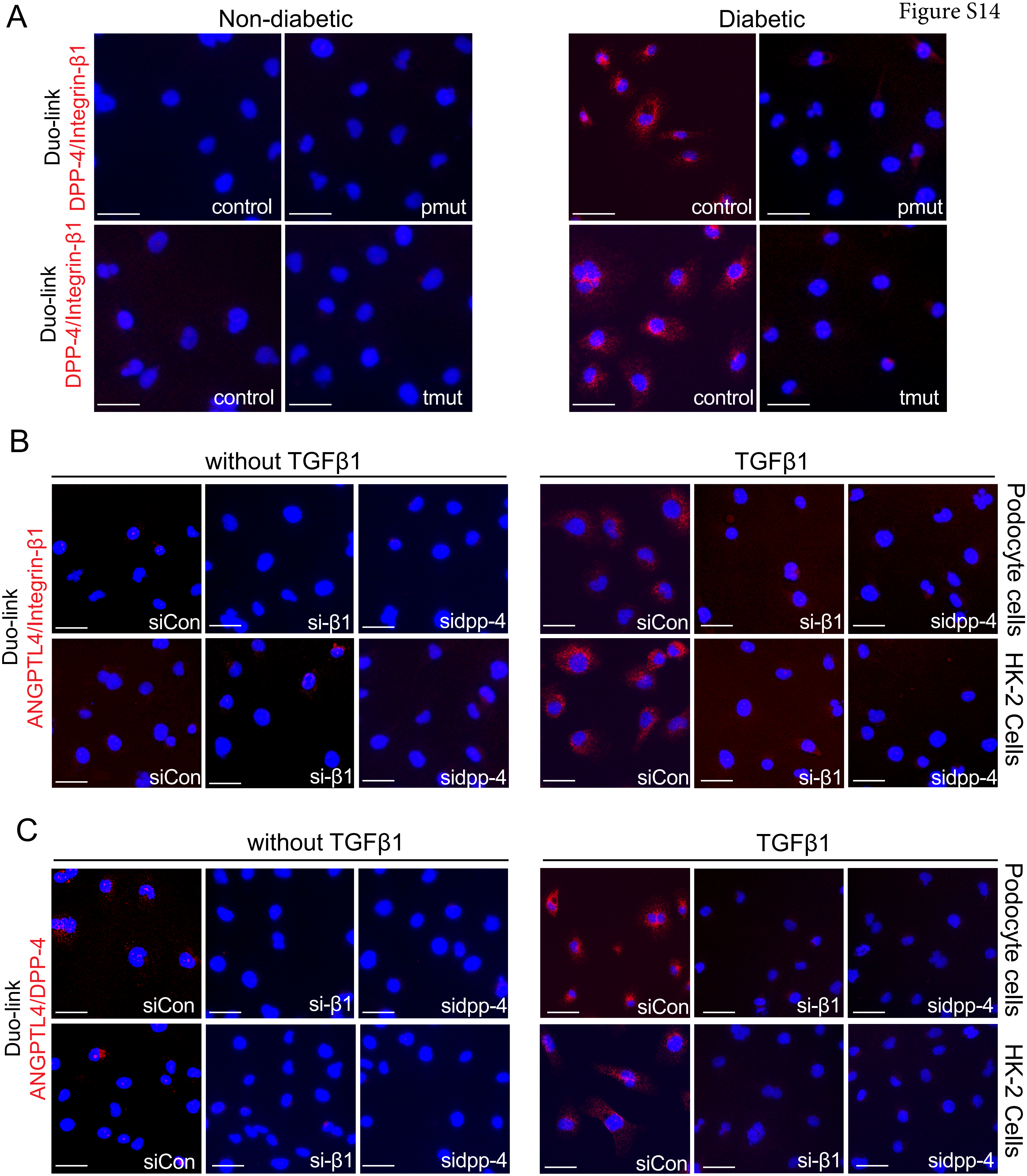
A
Non-diabetic
Diabetic
Duo-link
DPP-4/Integrin-β1
control
pmut
control
pmut
Duo-link
DPP-4/Integrin-β1
control
tmut
control
tmut
B
without TGFβ1
TGFβ1
Duo-link
ANGPTL4/Integrin-β1
siCon
si-β1
sidpp-4
Podocyte cells
HK-2 Cells
C
without TGFβ1
TGFβ1
Duo-link
ANGPTL4/DPP-4
siCon
si-β1
sidpp-4
Podocyte cells
HK-2 Cells

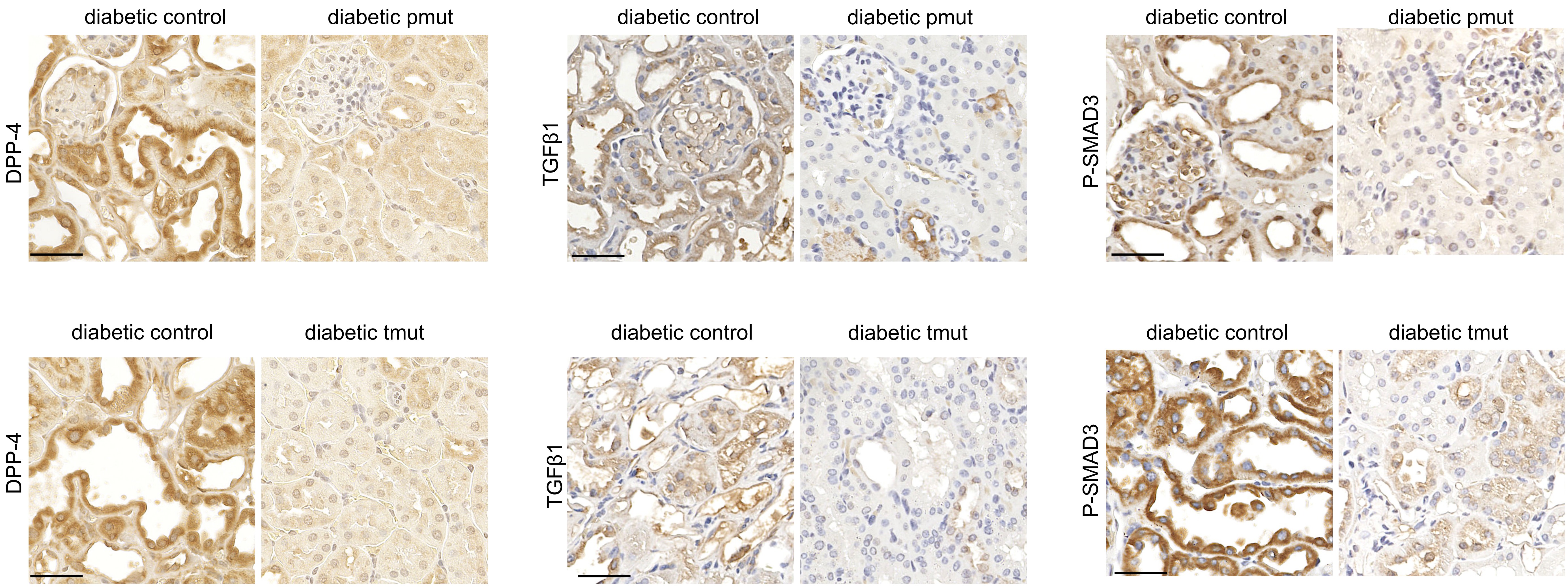

diabetic control
diabetic pmut
DPP-4
diabetic control
diabetic pmut
TGFβ1
diabetic control
diabetic pmut
P-SMAD3
diabetic control
diabetic tmut
DPP-4
diabetic control
diabetic tmut
TGFβ1
diabetic control
diabetic tmut
P-SMAD3

Figure S15

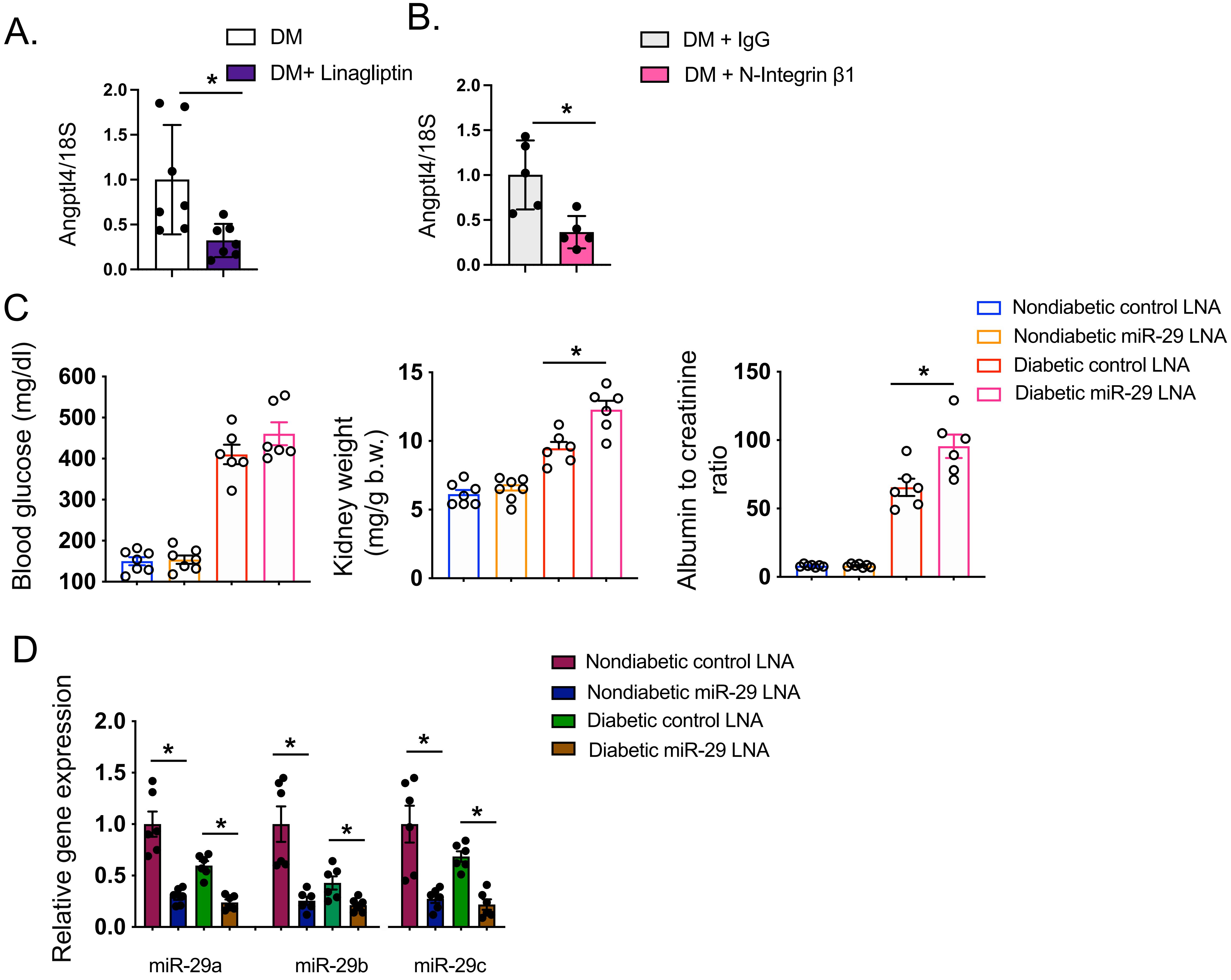
A.
DM
DM+ Linagliptin
Angptl4/18S
B.
DM + IgG
DM + N-Integrin β1
Angptl4/18S
C
Nondiabetic control LNA
Nondiabetic miR-29 LNA
Diabetic control LNA
Diabetic miR-29 LNA
Blood glucose (mg/dl)
Kidney weight (mg/g b.w.)
Albumin to creatinine ratio
D
Nondiabetic control LNA
Nondiabetic miR-29 LNA
Diabetic control LNA
Diabetic miR-29 LNA
Relative gene expression
miR-29a
miR-29b
miR-29c

Figure S16

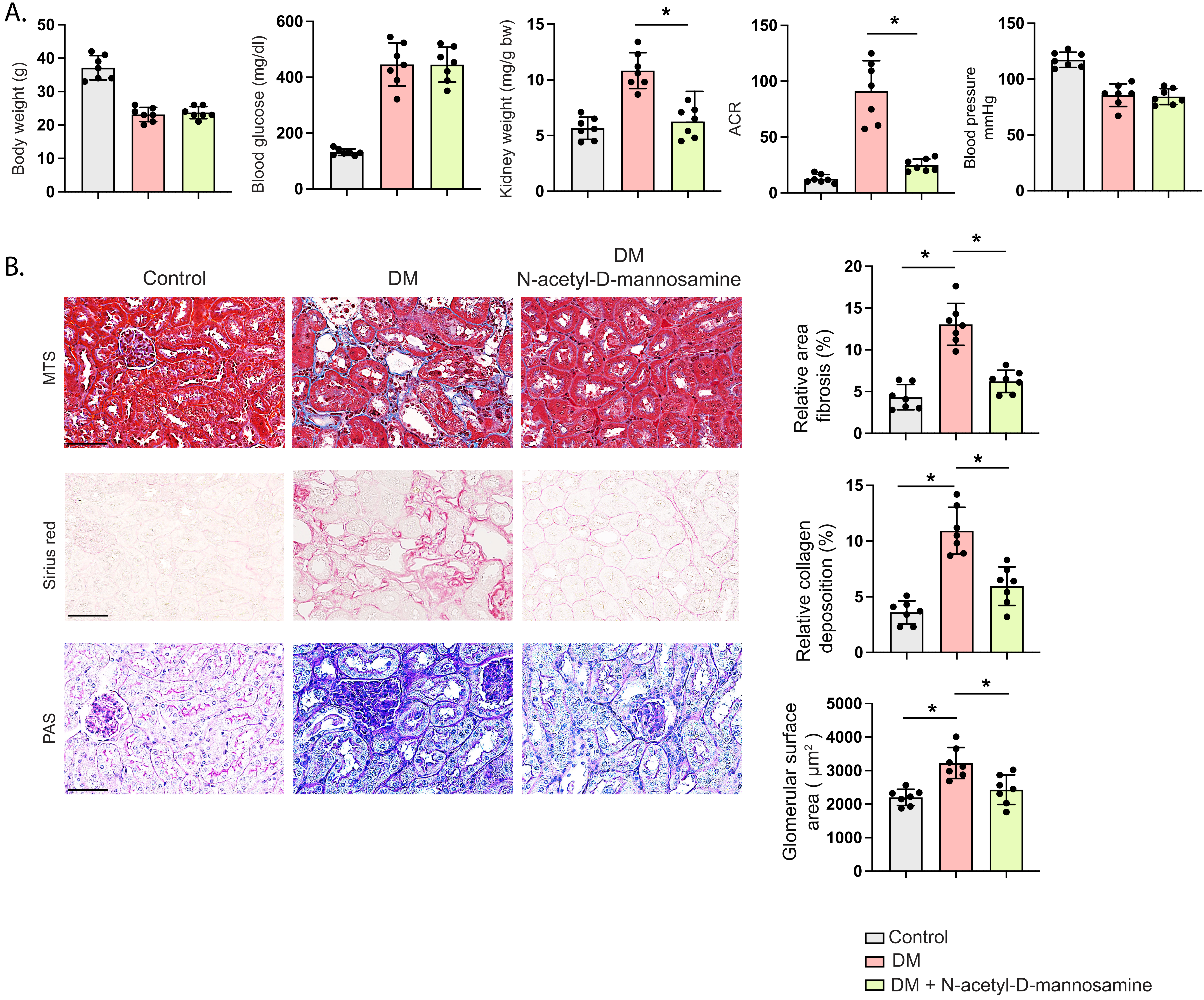
A.
Body weight (g)
Blood glucose (mg/dl)
Kidney weight (mg/g bw)
ACR
Blood pressure mmHg
B.
Control
DM
DM N-acetyl-D-mannosamine
MTS
Sirius red
PAS
Relative area fibrosis (%)
Relative collagen deposoition (%)
Glomerular surface area (μm²)
Control
DM
DM + N-acetyl-D-mannosamine

Figure S17

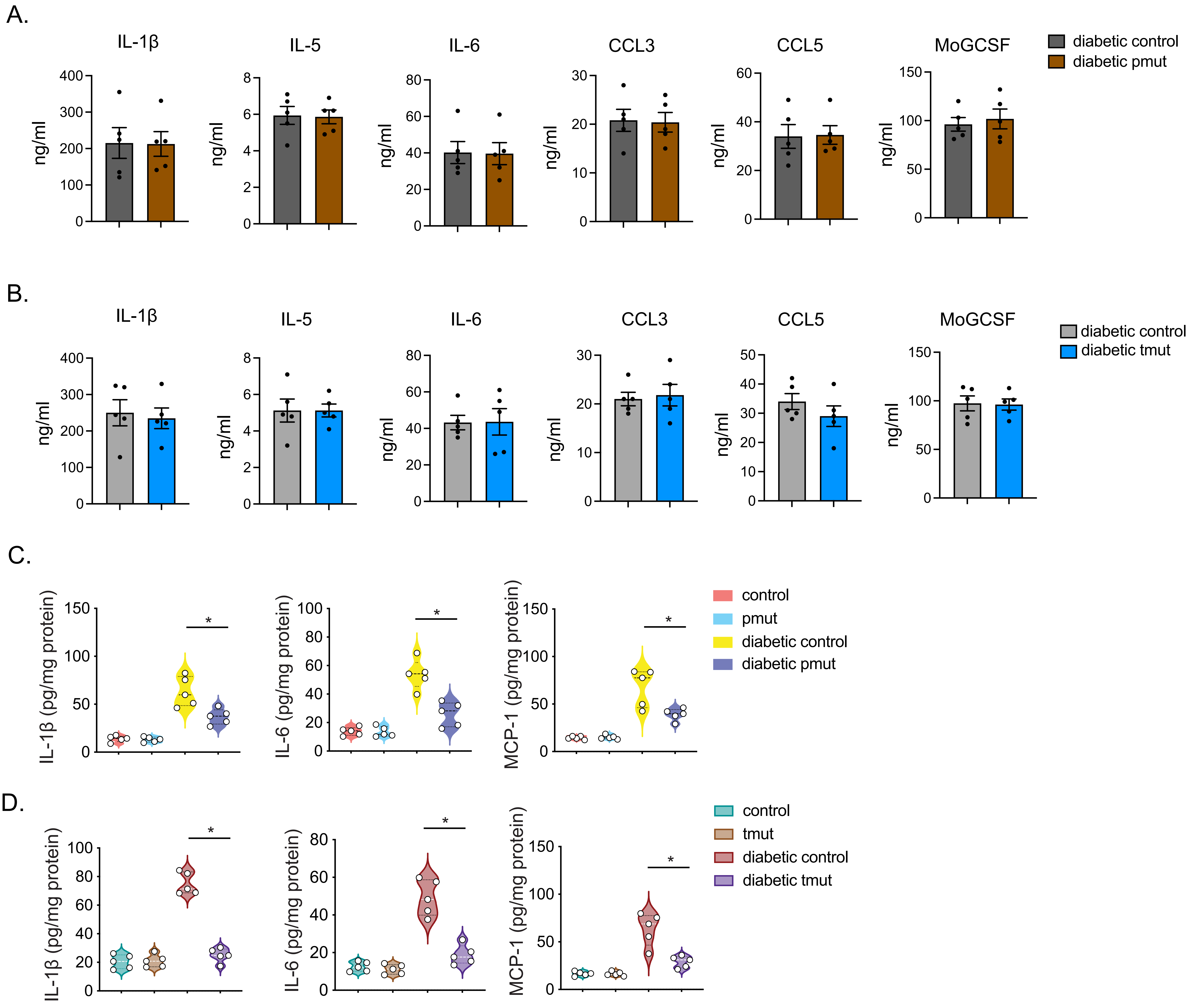
A.
IL-1β
IL-5
IL-6
CCL3
CCL5
MoGCSF
ng/ml
diabetic control
diabetic pmut
B.
IL-1β
IL-5
IL-6
CCL3
CCL5
MoGCSF
ng/ml
diabetic control
diabetic tmut
C.
IL-1β (pg/mg protein)
IL-6 (pg/mg protein)
MCP-1 (pg/mg protein)
*
control
pmut
diabetic control
diabetic pmut
D.
IL-1β (pg/mg protein)
IL-6 (pg/mg protein)
MCP-1 (pg/mg protein)
*
control
tmut
diabetic control
diabetic tmut

Figure S18

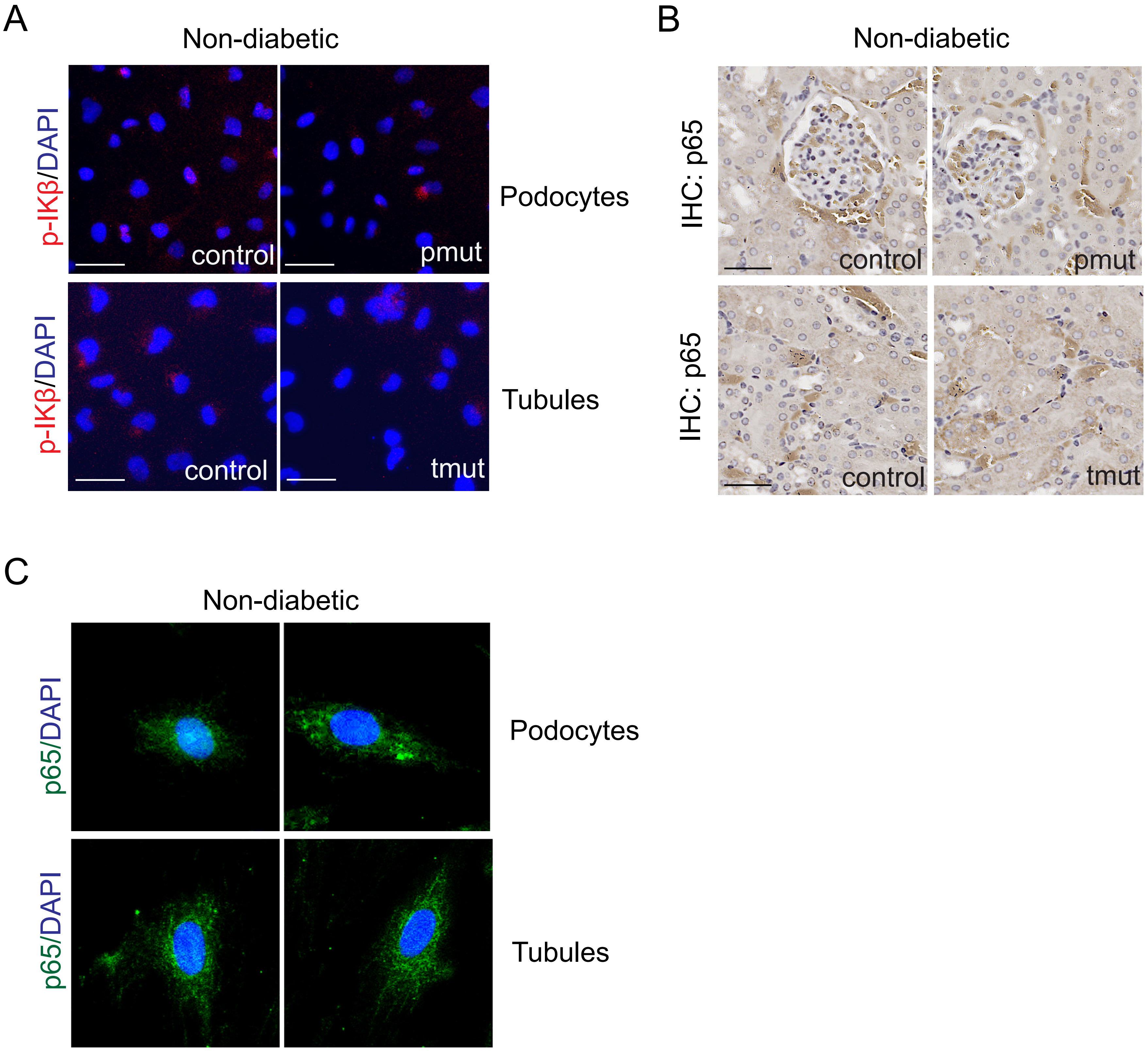
A
Non-diabetic
p-IKβ/DAPI
control
pmut
Podocytes
p-IKβ/DAPI
control
tmut
Tubules
B
Non-diabetic
IHC: p65
control
pmut
IHC: p65
control
tmut
C
Non-diabetic
p65/DAPI
Podocytes
p65/DAPI
Tubules

Figure S19

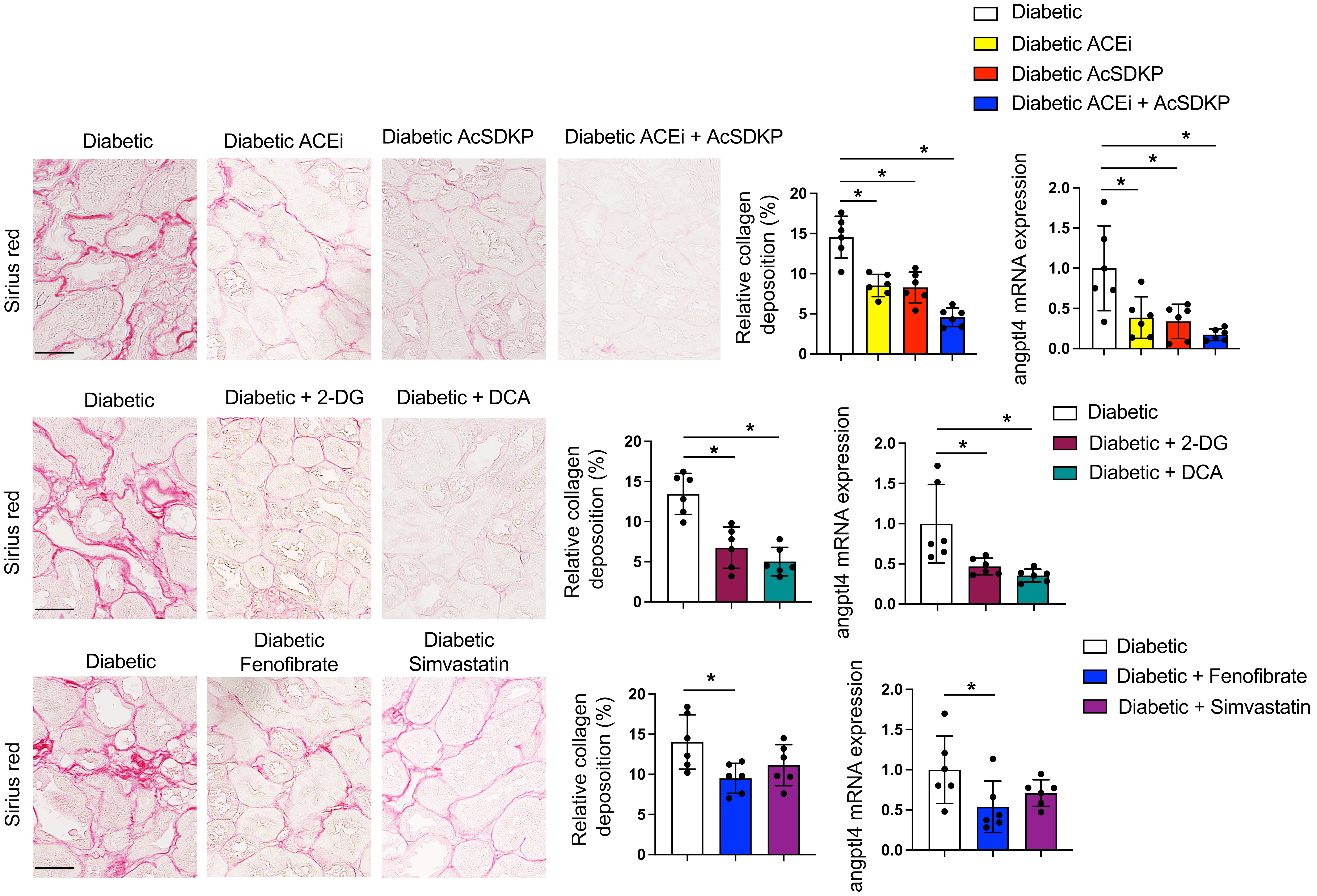

Diabetic
Diabetic ACEi
Diabetic AcSDKP
Diabetic ACEi + AcSDKP
Sirius red
Relative collagen deposoition (%)
angptl4 mRNA expression
Diabetic + 2-DG
Diabetic + DCA
Diabetic Fenofibrate
Diabetic Simvastatin
Diabetic + Fenofibrate
Diabetic + Simvastatin

Figure S20

Table S1: Sequences mouse primers used

Gene name	Forward primer	Reverse primer
TNFα	5′-AGGTCCCTACAGGGAACAAA	5′-TCTCTCTTTCCTTAGACACACGA
αSMA	5′-CTGACAGAGGCACCACTGAA	5′-GAAATAGCCAAGCTCAG
ANGPTL4	5'- ACTTCAGATGGAGGCTGGAC	5'- TCCGAAGCCATCCTTGTAGG
ANGPTL3	5'- GAGGAGCAGCTAACCAACTTAAT	5'- TCTGCATGTGCTGTTGACTTAAT
ANGPTL8	5'- GCTTTACACCTTCGAGCTGA	5'- ATCCAGGTAGTCTCAGGCTG
FSP-1	5'-TTCCAGAAGGTGATGAG	5'-TCATGGCAATGCAGGACAGGAAGA
Colla I	5'-ATCTCCTGGTGCTGATGGAC	5'- ACCTTGTTTGCCAGGTTCAC
IL-6	5′-TCTGAAGGACTCTGGCTTTG	5′-GATGGATGCTACCAAACTGGA
TGFβR1	5'- CGTGTGCCAAATGAAGAGGAT	5'- AAGGTGGTGCCCTCTGAAATG
Ndufv2	5'-TGGATGGCTACCTATCTCCGCT	5'-GGTACTTCCCAACTGGCTTTCG
Cpt1a	5′- GGTCTTCTCGGGTCGAAAGC	5′- TCCTCCCACCAGTCACTCAC
FASN	5'- CTTCGCCAACTCTACCATGG	5'-TTCCACACCCATGAGCGAGT
mt-Co1	5'- GCCCCAGATATAGCATTCCC	5'- GTTCATCCTGTTCCTGCTCC
mt-Cyb	5'-AGTAGACAAAGCCACCTTGA	5'-CCGCGATAATAAATGGTAAG
TFAM	5'- GAGCAGCTAACTCCAAGTCAG	5'- GAGCCGAATCATCCTTTGCCT
TMEM173	5'-TTTGCCATGTCACAGGATGC	5'-ATGAGGCGGCAGTTATTTCG
Mb21d1	5'-TGGTGGGAAGAGTGGTGATTTC	5'-TGCATTCCAATGGCAGAAGC
18S	5'-TTCCGATAACGAACGAGACTCT	5'-GGCTGAACGCCACTTGTC

Table S2: Sequences human primers

Gene name	Forward primer	Reverse primer
ANGPTL4	5'- CACAGCCTGCAGACACAACTC	5'-GGAGGCCAAACTGGCTTTGC
ANGPTL3	5'- CCTGAAACTCCAGAACACCCAG	5'- TTCCACGGTCTGGAGAAGGTCT
ANGPTL8	5'- CAGAAGGTGCTACGGGACAG	5'- AAATTCTCGGTAGGCAGGGC
IL-1β	5'-CCACAGACCTTCCAGGAGAATG	5'-GTGCAGTTCAGTGATCGTACAGG
IL-6	5'-AGACAGCCACTCACCTCTTCAG	5'-TTCTGCCAGTGCCTCTTTGCTG
TNFα	5'-CTCTTCTGCCTGCTGCACTTTG	5'-ATGGGCTACAGGCTTGTCACTC
18S	5'- CAGCCACCCGAGATTGAGCA	5'- TAGTAGCGACGGGCGGTGTG
